# Visual stimulation in automated gait rehabilitation for post-stroke patients

**DOI:** 10.3389/fbioe.2026.1769011

**Published:** 2026-05-28

**Authors:** Fu-Cheng Wang, Chia-Wei Chang, Hsin-Ti Cheng, Szu-Fu Chen, Yen-Chang Chien, Lik-Kang Koo, Lin-Yen Cheng

**Affiliations:** 1 Department of Mechanical Engineering, National Taiwan University, Taipei, Taiwan; 2 Department of Physical Medicine and Rehabilitation, Cheng Hsin General Hospital, Taipei, Taiwan; 3 Department of Physiology and Biophysics, National Defense Medical Center, Taipei, Taiwan

**Keywords:** gait, performance, rehabilitation, stroke, trainer, visual

## Abstract

This paper presents a visual stimulation system for an automated trainer employing neurodevelopmental treatment (NDT) principles. The traditional NDT can promote gait performance through repetitive tactile cues; however, its effectiveness is constrained by the need for continuous therapist involvement. Therefore, previous studies have presented automated gait trainers, which were capable of replicating tactile therapeutic interventions for gait rehabilitation. Recognizing the importance of visual perception in gait rehabilitation, we developed a visual stimulation system that incorporates both visual feedback training and action observation training to enhance rehabilitation outcomes. The visual system was then integrated with the NDT trainer to deliver synchronized tactile and visual cues during the rehabilitation sessions. We invited healthy individuals and post-stroke patients to participate in experiments and evaluated the rehabilitation effects. Based on the results, the visual stimulation system is shown to be effective in improving gait performance.

## 1 Introduction

Stroke and other cerebrovascular disorders are the third leading cause of mortality worldwide (World Health Organization, 2025). The burdens imposed by these disorders arise in part due to modern lifestyles, which are characterized by high psychosocial stress and poor attention to physical health, leading to harmful behavioral patterns. In the case of stroke, its effects extend beyond its acute neurological insults to frequently precipitate persistent functional deficits. These deficits most notably affect locomotor capacities, thereby profoundly compromising autonomy in activities of daily living. These adverse stroke outcomes highlight a pressing need for effective rehabilitation strategies aimed at restoring post-stroke locomotor function to enhance the stroke survivors’ quality of life.

This need is now being extensively addressed through investigations exploring technological interventions aimed at post-stroke gait rehabilitation. For example, Roy et al. (2007) developed a wearable robotic system for ankle rehabilitation, while Colombo et al. (2000) integrated a treadmill-based system with a robotic exoskeleton to assist with gait training in subjects with foot paralysis. Similarly, Yano et al. (2010) proposed a gait training device to enhance walking stability and pacing on uneven terrains. These types of approaches have demonstrated promising therapeutic benefits; however, their underlying strategies rely predominantly on mechanically guiding patients through predetermined movement trajectories. Thus, they emphasize external physical interventions to train structured motor patterns rather than promoting adaptive neuromotor recovery.

In contrast, neurodevelopmental treatment (NDT) represents a therapeutic paradigm that prioritizes the enhancement of gait performance, postural stability, and overall physical mobility with minimal physical intervention. Empirical evidence supports the efficacy of NDT in improving gait patterns in post-stroke individuals with hemiparesis (Mikolajewska, 2013), and its applicability extends to mitigating locomotor impairments in other neurological conditions, such as cerebral palsy (Kalaichandran and Swarnakumari, 2019). These findings further support NDT as a promising and evidence-based training approach for improving gait functions within stroke rehabilitation frameworks (Pathak, 2021). In conventional NDT practice (Visintin et al., 1998), one therapist stands behind the patient to guide pelvic motion and facilitate proper weight shifting, while another therapist positions beside the paretic limb to assist stepping and control of the limb during both the stance and swing phases. Consequently, NDT-based rehabilitation is highly labor-intensive, and patients often receive inadequate training due to the limited availability of therapist support.

Recent research has focused on automating NDT-based rehabilitation. For instance, Wang et al. (2020) developed an automated NDT trainer that can emulate the therapeutic tactile cues that were applied during traditional NDT interventions. Observations from manual NDT sessions revealed that therapists typically apply tactile stimulation to the patient’s anterior superior iliac spine (ASIS) when a heel strike (HS) is detected on the contralateral side. Therefore, accurate and timely HS detection has become a critical prerequisite for implementing automated NDT protocols.

This recognition of the importance of HS events in gait rehabilitation has spurred extensive research on gait pattern detection and analysis. For instance, Aminian et al. (2002) proposed a system capable of extracting spatiotemporal gait parameters through miniature gyroscopes. Yun et al. (2012) addressed drift correction in position estimation by integrating acceleration data with inertial and magnetic sensor measurements. Rebula et al. (2013) developed algorithms for estimating foot placement and gait variability using inertial sensors. Wang et al. (2020) employed visual recognition techniques to detect HSs by an NDT trainer. Wang et al. (2021) built a long short-term memory (LSTM) model that used inertial measurement unit (IMU) data to perform HS recognition.

These advancements underscore the growing integration of computer vision and machine learning approaches in automated gait event detection. The experimental results have demonstrated that integrating gait recognition technologies into NDT-based trainers can improve gait performance (Wang et al., 2020; Shih et al., 2023). However, these previous implementations of automated NDT training have primarily focused on delivering tactile stimuli at the HS events to facilitate gait rehabilitation and have not focused on integrating visual perception with motor function, although coordinating vision and motor activity would enable real-time feedback from the external environment, thereby improving spatial awareness and motor coordination. Evidence suggests that visual stimulation training can enhance perceptual processing and motor control, making it particularly beneficial for individuals with neurological impairments, including those recovering from stroke (Pazzaglia and Galli, 2015). In addition, emerging technologies, such as augmented reality (Pirovano et al., 2016) and virtual reality (Felipe et al., 2020), can amplify visual stimuli, thereby offering immersive and interactive rehabilitation experiences. These approaches can increase patients’ engagement and motivation while also contributing to more effective and sustainable rehabilitation outcomes.

In post-stroke patients, the combined use of gait training and visual biofeedback has been shown to improve functional outcomes (Kazmierczak et al., 2022). Visual perception plays a critical role in gait rehabilitation by facilitating visuomotor adaptation and observational learning, processes that are mechanistically distinct from kinesthetic feedback. Visual feedback training (VFT) promotes error-based learning, in which real-time discrepancies between intended and actual movements generate corrective signals that drive adaptation within cerebellar–parietal circuits (Rabe et al., 2009; Donchin et al., 2012). In contrast, action observation training (AOT) engages mirror neuron networks to enhance corticospinal excitability and motor imitation, even in the absence of explicit performance errors (Kang et al., 2012; Zhang et al., 2019). These visual mechanisms are neuroanatomically and functionally separable from tactile and proprioceptive processing, thereby providing a foundation for multimodal synergy. When synchronized with tactile cues, visual inputs may compensate for post-stroke sensory deficits and strengthen sensorimotor mapping, ultimately leading to improved gait symmetry and postural stability (Moore and Cluff, 2021).

Recent multimodal studies support this additive effect, demonstrating that visual–tactile integration outperforms single-modality interventions in improving balance (Shin and Chung, 2022) and motor performance (Kim et al., 2025). Specifically, Shin and Chung (Shin and Chung, 2022) reported that synchronized visual and rhythmic cues enhanced balance timing and coordination, whereas Kim et al. (2025) demonstrated improved gait symmetry associated with cross-modal sensory integration between visual and somatosensory pathways. Together, these findings provide a mechanistic basis for combining visual stimulation with NDT-based tactile cueing to enhance post-stroke gait rehabilitation. Building on this evidence, this paper proposes the delivery of synchronized visual stimulation and tactile cues to post-stroke subjects using an NDT trainer with a speed-adaptive treadmill (Wang et al., 2025) as a means of promoting patient engagement and therapeutic outcomes. Unlike conventional approaches that deliver visual or tactile stimuli in isolation, our system provides concurrent multimodal cues to improve gait performance and enhance rehabilitation efficacy. Accordingly, this study tests the following hypothesis: that synchronized VFT or AOT with NDT tactile cues will enhance gait performance more than tactile cues alone.

The aim of this study was to extend the progress in NDT by incorporating a visual stimulation system into an NDT trainer and to evaluate the effects on rehabilitative performance. Here, we developed an intuitive visual stimulation interface, which assists patients in improving their gait performance through either VFT or AOT. The visual stimulation system was integrated into a stationary NDT trainer to deliver both tactile and visual cues. We invited healthy individuals and post-stroke patients to conduct clinical experiments. The results demonstrated that incorporating visual cues into NDT training can improve gait performance in terms of longitudinal symmetry, pelvic rotation, walking speed, and stride length.

## 2 Methods

### 2.1 The NDT trainer

**FIGURE 1:**
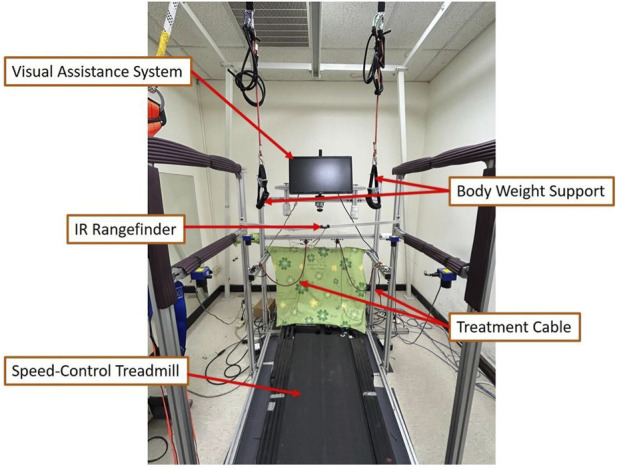
The NDT trainer.

The NDT trainer (Figure 1) comprises five subsystems: a motion capture system, an expert system, a cable control system, a treadmill control system, and a visual stimulation system. Detailed specifications of these components are given in Supplementary Table S1 in the Supplementary Material.

The motion capture system employs the APDM Opal system (Apdm, Inc, 2025; Wang et al., 2025) with three IMUs to measure the subjects’ kinematic data. Each IMU captures tri-axial linear acceleration and tri-axial angular velocity signals at a sampling rate of 25 Hz. One IMU was attached to the participant’s waist to measure pelvic rotation, while two IMUs were mounted on the lower limbs to detect HS events using a LSTM model trained on kinematic signals (Wang et al., 2021). The detected HS events subsequently trigger the expert system, cable-control system, and visual stimulation system. The expert system processes real-time IMU data to determine the timing for intervention, whereas the cable control system delivers corresponding tactile stimulation to the subject’s ASIS through the cable control system (Wang et al., 2020). The treadmill control system incorporates an infrared range sensor to track user movement and automatically adjust the motor output, ensuring that the user remains within the designated walking zone (Wang et al., 2025). The visual stimulation system provides interactive images according to the user’s walking patterns, enhancing user engagement and facilitating gait correction through intuitive visual cues.

**FIGURE 2:**
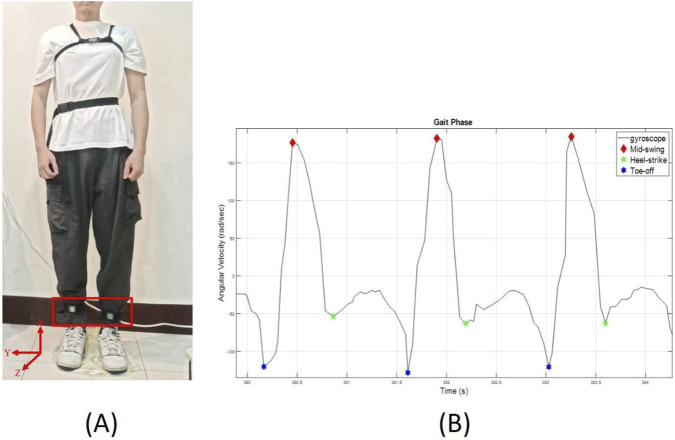
Gait measurement and patterns (Wang et al., 2021). **(A)** IMU attachments. **(B)** Gait responses.

Accurate HS detection is critical for initiating NDT interventions, as the human gait is characterized as a cyclic pattern consisting of the mid-swing (MS), HS, and toe-off phases (Pirker and Katzenschlager, 2017). Previous clinical studies have reported that therapists usually apply tactile cues to each subject’s ASIS upon identifying the HS events (Wang et al., 2020). To automate this process, we attached two IMUs to the subject’s shanks, as illustrated in Figure 2A, to capture lower-limb kinematic data. Figure 2B illustrates an example of sagittal-plane angular velocity, where peak values correspond to MS phases and the subsequent troughs indicate HS events. To enable real-time HS recognition and automatic triggering of NDT interventions during gait rehabilitation, we trained a LSTM model following the methodology described in (Wang et al., 2021). The model development consisted of two phases: a training phase and a validation phase, yielding an average positive predictive value exceeding 98% and an average mean error of less than 0.04 s. In the present study, this LSTM model was integrated into the experimental protocol to perform HS recognition in real time.

### 2.2 Rehabilitation intervention

We implemented the NDT intervention protocols (Wang et al., 2020; Shih et al., 2023), whereby the cable control system delivers forces synchronized with HS events, as Equation 1:

$$F\left(t\right)=\frac{\left(MF_{\max}-F_{\min}\right)}{2}\times \sin\left(2\pi f\right)+\frac{\left(MF_{\max}+F_{\min}\right)}{2}$$ (1)

The force command was constrained within predefined limits, ranging from a minimum of $F_{\min}$ = 1 lb. to a maximum of $F_{\max}$ = 6 lb. with a frequency of *f* = 1 Hz. When the pelvic rotation fell below a threshold of 12^o^, the intervention force was amplified by a factor of *M* = 1.5 to enhance therapeutic efficacy.

### 2.3 Visual stimulation system

Visual stimulation, such as VFT and AOT, is widely employed in neurorehabilitation to promote motor control and functional recovery. VFT provides real-time, dynamic visual feedback, enabling individuals to adjust their gait or posture based on visual cues. By observing their own movements, patients can actively correct errors, thereby improving accuracy and coordination. For example, VFT has been applied to improve balance in stroke patients (Cikajlo et al., 2020) and incorporated into gait training to enhance walking symmetry (Kim and Kim, 2020).

In contrast, AOT utilizes the mirror neuron system by allowing patients to observe and imitate others’ movements. Studies have shown that combining mirrored observation with task-oriented therapy can enhance corticospinal excitability, reduce muscle response time, and improve motor performance (Kang et al., 2012). Patients may also be guided to mimic mirrored movements of the nonaffected limb (Roberts et al., 2021).

**FIGURE 3:**
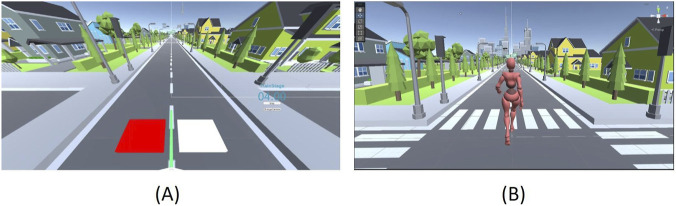
The visual stimulation system. **(A)** Flashing square cues. **(B)** Animated stepping cues.

Visual stimuli can deliver real-time feedback through dynamic graphics or animations to reinforce correct movement patterns and enhance gait performance. Meta-analytic evidence indicates that visual feedback can facilitate motor recovery and improve movement precision in individuals after stroke (Zhang et al., 2019; Peng et al., 2021). In this study, we developed two visual stimulation methods: flashing square cues and animated stepping cues. The flashing square cue is VFT-based and is designed to improve gait rhythm and coordination through immediate visual reinforcement. As shown in Figure 3A, two squares represent the left and right feet. The left square turns red when the right HS is detected, and *vice versa*. This synchronization promotes symmetrical swing phases, providing intuitive feedback to help users perceive gait patterns in real time.

Conversely, the animated stepping cue is AOT-based and employs human-like avatars to simulate leg movements. As illustrated in Figure 3B, the avatar raises the right leg upon detecting a left HS, and *vice versa*. By observing these motions, users are encouraged to imitate correct gait patterns through repeated visual–motor interaction.

### 2.4 Experiments

We recruited ten healthy individuals and twenty post-stroke patients to participate in clinical experiments. The subjects’ demographic data are presented in Supplementary Tables S2–S4 in the Supplementary Material. The healthy group had a mean age of 25.1 years (standard deviation: 4.23), whereas the post-stroke group had a mean age of 69.2 years (standard deviation: 7.28). To simulate a hemiparetic gait, healthy subjects wore a gaiter and ankle weights to restrict knee flexion and limit limb control and joint mobility. All healthy participants completed both experiments with flashing square and animated stepping cues. The post-stroke patients, classified as Brunnstrom Stage III–V, were randomly divided into two groups: ten received training with flashing square cues, and ten with animated stepping cues. Participants were excluded if they had a history of significant visual impairments, including visual field defects or hemispatial neglect. All subjects had normal or corrected-to-normal vision, as verified by medical history and a pre-training functional assessment. All procedures were approved by the Institutional Review Board of Cheng Hsin General Hospital (IRB number: (1140)113-70), and written informed consent was obtained from all participants.

Prior to training, a familiarization phase was conducted during which participants observed the stimuli from the experimental viewing distance while the instructors introduced the experimental procedures. This functional assessment ensured that all subjects possessed the necessary visual acuity to respond to the cues, thereby minimizing visual impairment as a potential confounder. The participants then walked a few steps on the treadmill to become familiar with the speed-control mechanism.

During the formal experiments, participants initially stood steadily on the treadmill, which was driven by a motor with a maximum speed of 800 rpm. For safety considerations, an upper speed limit of 476 rpm was imposed, corresponding to a belt speed of approximately 0.54 m/s after the gear reduction mechanism. Participants then began walking at their self-selected pace, while the speed-control module continuously adjusted the treadmill speed to maintain an approximate distance of 30 cm in front of the infrared range sensor. Each subject completed five experimental stages:

1. Stage *A*: walking for 50 s on a treadmill without external intervention.
2. Stage *B*: walking for 150 s with tactile intervention.
3. Stage *D*: walking for 200 s with combined tactile and visual cues.
4. Stage $\bar{B}$ : Walking for 150 s with only tactile cues.
5. Stage $\bar{A}$ : Walking for 50 s without any external intervention.

Each participant completed five consecutive experimental stages without scheduled rest periods; however, breaks were allowed upon request. Owing to the speed-control mechanism, the treadmill automatically stopped when participants ceased walking. A medical doctor was present throughout the experiment and had the authority to terminate the session immediately if signs of fatigue or discomfort were observed. All participants successfully completed the experiments, and no discernible effects of resting on performance were observed.

The effectiveness of visual intervention was evaluated by comparing the following rehabilitation outcomes across stages: longitudinal symmetry, pelvic rotation, walking speed, and stride length.

### 2.5 Performance evaluation

The effectiveness of stroke rehabilitation was assessed using four performance indexes: longitudinal symmetry, pelvic rotation, walking speed, and stride length.

1. Longitudinal symmetry: Longitudinal symmetry is a key indicator for evaluating forward walking patterns. It is defined as the ratio of swing phases between the two limbs, as in Equation 2:

$$Asym_{SP}=\frac{SP_{P}-SP_{NP}}{SP_{P}}\times 100\%$$ (2)

where $SP_{P}$ and $SP_{NP}$ denote the swing phases of the paretic and non-paretic limbs, respectively. The swing phase is defined as in Equation 3:

$$SP=\frac{T_{SW}}{T_{GAIT}}\times 100\%$$ (3)

where $T_{SW}$ denotes the swing time, which is the interval from toe-off to the subsequent HS, while $T_{GAIT}$ represents the gait time from 1 HS to the next HS on the same foot. Healthy individuals typically exhibit bilateral symmetry ( $Asym_{SP}\approx 0$ ), whereas post-stroke patients often show gait asymmetry due to impaired strength and stability on the affected side (Wall and Turnbull, 1986). Rehabilitation is considered effective when $Asym_{SP}$ decreases after the training.

2. Pelvic rotation: Pelvic rotation is an important clinical parameter for assessing gait stability and rehabilitation progress. It is defined as in Equation 4:

$${Amp}_{\mathrm{PR}}=\theta_{\max}-\theta_{\min}$$ (4)

where $\theta_{\max}$ and $\theta_{\min}$ represent the maximal and minimal pelvic rotational angles occurring between two consecutive HS events. Post-stroke patients typically exhibit reduced pelvic rotation because of impaired muscle strength, which compromises their dynamic stability. Rehabilitation is considered effective when ${Amp}_{\mathrm{PR}}$ increases after the training.

3. Walking speed: Walking speed is also a key indicator of post-stroke functional mobility, as it reflects the integrated performance of neuromuscular, cardiovascular, and balance systems (Capo-Lugo et al., 2012; Grau-Pellicer et al., 2019). Stroke survivors generally walk more slowly than healthy individuals, and their reduced speed is associated with impaired community mobility, increased fall risk, loss of independence, and lower quality of life (Tasseel-Ponche et al., 2022).

Standardized assessments, such as the 6-Minute Walk Test and the 10-m Walk Test, are usually applied to evaluate walking speed (Lauziere et al., 2016). Research has shown that improvements in walking speed correlate with better balance, gait consistency, and dynamic stability (Capo-Lugo et al., 2012). Walking speed can also serve as a predictor of hospitalization duration, discharge planning, and long-term ambulatory outcomes (Tasseel-Ponche et al., 2022; Jasper et al., 2025). Rehabilitation is effective when the subjects’ walking speed increases after the training.

4. Stride length: Stride length is defined as the length between two consecutive HSs on the same limb and represents a critical spatiotemporal parameter in gait analyses. Post-stroke gait often exhibits stride length asymmetry, in which the paretic limb produces shorter strides than the nonparetic limb. Such reductions in stride length reflect impairments in propulsion, swing-phase coordination, and balance control, all of which contribute to decreased gait efficiency, elevated energy expenditure, increased fall risk, and prolonged rehabilitation duration (Schwartz et al., 2022; Jasper et al., 2025).

In some cases, stride lengths represent a compensatory adaptation. For instance, reduced hip-flexor activation on the paretic side may result in excessive reliance on the contralateral limb to advance the body forward (Allen et al., 2011). Because stride length strongly influences walking speed, limitations in stride extensibility often leads to compensatory increases in cadence, which reduce energy efficiency and hinder gait symmetry (Ettema et al., 2025). Rehabilitation is considered effective when stride length increases after training.

## 3 Results

Two clinical experiments that employed distinct visual cueing strategies were conducted: the flashing square cue and the animated stepping cue. Initially, ten healthy participants took part in experiments to verify system safety and demonstrate the potential benefits for gait rehabilitation. Based on the preliminary findings, post-stroke patients were subsequently recruited to undergo NDT gait training incorporating both tactile and visual cues. We collected the experimental data and calculated the performance metrics for analysis and comparison.

### 3.1 Flashing square cues

Performance indexes from experiments employing flashing square cues are presented in Supplementary Figures S1, S2 and Supplementary Tables S4, S5 in the Supplementary Material. The results indicate that NDT training augmented with integrated visual and tactile cues can produce consistent improvements, including reduced longitudinal asymmetry and increased pelvic rotation, walking speed, and stride length.

The performance indexes of healthy participants are summarized in Table 1. First, all participants demonstrated improvements across all training stages. Among them, six achieved their greatest improvements at stage *D*. The average improvements were 37.72%, 34.63%, and 28.39% at stages *D*, $\bar{B}$ , and $\bar{A}$ , respectively, highlighting the effectiveness of visual cues in enhancing gait symmetry. Most improvements occurred during visual cue application, with some sustained effects observed at later stages. Specifically, one, four, and four participants reached their highest improvements in pelvic rotation at stages *B*, *D*, and $\bar{A}$ , respectively. The average improvements were 23.93%, 18.95%, and 23.13% at stages *D*, $\bar{B}$ , and $\bar{A}$ , respectively, underscoring the positive impacts of visual cues on pelvic rotation and stability. All participants improved their walking speeds at all stages, with 100% achieving their peak performance at stages *D* and $\bar{B}$ , which were identical due to the speed limitation of the treadmill. The average improvements were 144.86%, 173.41%, 173.41%, and 170.88%, at stages *B*, *D*, $\bar{B}$ , and $\bar{A}$ , respectively, confirming the efficacy of visual cues for increasing walking speed. Finally, nearly all participants improved their stride length across the stages, except for subject H1 at stage *B*. Among the participants, four achieved their greatest improvements at stages *D* and $\bar{A}$ .

**TABLE 1:** Performance indexes and improvements with flashing square cues (healthy subjects).

Subject	A	B	D	$\bar{B}$	$\bar{A}$
Average $Asym_{SP}$ (%) (imp%)					
H1	22.34	13.67 (38.80)	11.61 (48.04)	11.61 (48.03)	14.53 (34.98)
H2	15.92	15.00 (5.81)	10.00 (37.21)	13.94 (12.46)	15.88 (0.27)
H3	11.88	11.74 (1.18)	10.52 (11.47)	9.67 (18.62)	11.63 (2.16)
H4	21.54	17.54 (18.54)	16.92 (21.44)	16.96 (21.26)	17.42 (19.11)
H5	37.98	23.94 (36.96)	13.88 (63.45)	15.97 (57.94)	20.05 (47.21)
H6	10.64	7.11 (33.16)	5.79 (45.59)	5.60 (47.38)	5.80 (45.52)
H7	16.85	13.73 (18.55)	7.76 (53.97)	8.21 (51.28)	7.73 (54.12)
H8	13.88	13.63 (1.83)	11.81 (14.87)	10.86 (21.74)	11.28 (18.70)
H9	13.62	10.16 (25.44)	7.08 (48.00)	7.72 (43.30)	9.63 (29.32)
H10	20.63	16.71 (19.01)	13.78 (33.24)	15.62 (24.29)	13.90 (32.65)
Average $Amp_{\mathrm{PR}}$ (o) (imp%)					
H1	8.01	9.51 (18.67)	16.83 (110.02)	13.94 (73.96)	16.63 (107.53)
H2	14.81	14.37 (−2.97)	8.22 (−44.48)	7.15 (−51.68)	7.21 (−51.31)
H3	8.15	7.14 (−12.38)	8.97 (10.02)	7.17 (−12.07)	4.84 (−40.62)
H4	8.28	11.85 (43.14)	15.57 (88.09)	12.61 (52.35)	9.09 (9.79)
H5	14.92	12.15 (−18.56)	17.76 (19.05)	22.43 (50.38)	23.75 (59.22)
H6	9.52	8.48 (−10.93)	10.16 (6.75)	12.21 (28.27)	14.95 (57.03)
H7	5.17	6.17 (19.22)	6.31 (21.95)	6.67 (28.84)	7.36 (42.24)
H8	10.73	11.19 (4.21)	11.52 (7.28)	11.33 (5.53)	11.42 (6.40)
H9	5.86	7.69 (31.20)	6.10 (4.13)	5.94 (1.40)	6.40 (9.11)
H10	8.45	9.68 (14.54)	9.83 (16.34)	9.49 (12.38)	11.12 (31.57)
Average walking speed (rpm) (imp%)					
H1	137.9	377.6 (173.77)	476.31 (245.34)	476.31 (245.34)	476.31 (245.34)
H2	129.5	438.4 (238.60)	476.31 (267.89)	476.31 (267.74)	443.6 (242.67)
H3	419.2	476.31 (13.63)	476.31 (13.63)	476.31 (13.63)	476.31 (13.63)
H4	200.2	469.2 (134.39)	476.31 (137.96)	476.31 (137.96)	476.31 (137.96)
H5	269.6	396.5 (47.04)	476.31 (76.65)	476.31 (76.65)	476.31 (76.65)
H6	108.0	401.7 (271.80)	476.31 (340.85)	476.31 (340.85)	476.31 (340.85)
H7	162.0	401.9 (148.03)	476.31 (193.95)	476.31 (193.95)	476.31 (193.95)
H8	283.6	452.2 (59.44)	476.31 (67.92)	476.31 (67.92)	476.31 (67.93)
H9	132.8	452.4 (240.58)	476.31 (258.62)	476.31 (258.62)	476.31 (258.62)
H10	206.2	455.7 (121.06)	476.31 (131.05)	476.31 (131.05)	476.31 (131.05)
Average stride length (cm) (imp%)					
H1	87.86	83.95 (−4.44)	101.25 (15.25)	99.95 (13.76)	101.37 (15.38)
H2	57.26	108.33 (89.20)	68.91 (20.35)	61.50 (7.41)	58.12 (1.5)
H3	68.67	69.65 (1.42)	81.38 (18.50)	77.24 (12.48)	74.78 (8.9)
H4	55.56	90.17 (62.30)	97.08 (74.74)	95.86 (72.54)	92.70 (66.85)
H5	65.56	75.49 (15.15)	77.92 (18.86)	85.49 (30.40)	87.06 (32.8)
H6	58.40	83.19 (42.22)	78.36 (34.17)	86.31 (47.79)	89.31 (52.92)
H7	38.54	58.61 (52.10)	64.88 (68.37)	65.01 (68.69)	74.33 (92.88)
H8	70.93	85.13 (20.02)	89.70 (26.46)	77.03 (8.61)	78.98 (11.35)
H9	39.64	62.20 (56.92)	65.06 (64.15)	62.23 (57.01)	63.03 (59.01)
H10	48.81	69.94 (43.28)	65.20 (33.58)	61.91 (26.84)	63.84 (30.79)

imp%: percentage improvement compared with Stage *A*.

Given the confirmed safety and promising rehabilitation effects observed in the healthy participants, clinical experiments were conducted with ten post-stroke patients. Their performance indexes are shown in Supplementary Figure S2 and are summarized in Table 2. All post-stroke patients exhibited improvements in $Asym_{SP}$ at all stages, and six subjects achieved their greatest gains at stage *D*. The average improvements were 37.72%, 34.63%, and 28.39% at stages *D*, $\bar{B}$ , and $\bar{A}$ , respectively, demonstrating both immediate and delayed effects of visual cues on longitudinal symmetry. Nine patients showed improved ${Amp}_{\mathrm{PR}}$ across stages *D*, $\bar{B}$ , and $\bar{A}$ (the exception was subject S5). Of those nine, six reached their peak improvement at stage $\bar{B}$ , showing the sustained effects of visual cues on pelvic rotation. The average improvements at stages $\bar{B}$ and $\bar{A}$ were 38.74% and 38.56%, respectively, indicating significant and sustained effects of visual cues on pelvic rotation. The patients walked at speeds comparable to those of the healthy subjects at stage *A*, but generally at lower speeds than the healthy subjects after the intervention, possibly due to physical limitations. The walking speed increased across all patients and stages, and seven patients reached their highest improvements at stage $\bar{B}$ , suggesting delayed effects on speed improvement. Because of speed limitations, some patients achieved the highest speed at multiple stages (e.g., subject S5 achieved the maximum speed at stages *B*, *D*, $\bar{B}$ , and $\bar{A}$ ). The average improvements were 82.94% and 86.69% at stages $\bar{B}$ and $\bar{A}$ , again suggesting delayed effects on walking speed. Six patients achieved their largest stride length at stage *D*, confirming the positive impact of visual cues on stride enhancement.

**TABLE 2:** Performance indexes and improvements with flashing square cues (stroke patients).

Subject	A	B	D	$\bar{B}$	$\bar{A}$
Average $Asym_{SP}$ (%) (imp%)					
S1	19.53	16.50 (15.53)	15.83 (18.94)	16.27 (16.69)	18.06 (7.55)
S2	33.86	24.18 (28.60)	23.64 (30.19)	23.23 (31.40)	26.28 (22.38)
S3	40.46	28.25 (30.18)	26.44 (34.66)	26.50 (34.49)	32.15 (20.54)
S4	17.80	11.83 (33.51)	9.32 (47.63)	9.62 (45.93)	9.09 (48.92)
S5	10.82	10.80 (0.20)	9.68 (10.58)	9.18 (15.14)	9.13 (15.61)
S6	35.16	26.30 (25.20)	17.08 (51.43)	19.56 (44.38)	14.00 (60.18)
S7	20.51	17.16 (16.30)	9.70 (52.71)	17.16 (16.34)	14.55 (29.04)
S8	34.82	26.46 (24.02)	24.16 (30.61)	24.42 (29.87)	29.76 (14.54)
S9	24.03	20.92 (12.94)	17.41 (27.55)	21.04 (12.46)	23.10 (3.88)
S10	40.29	38.15 (5.32)	36.68 (8.98)	37.32 (7.38)	39.11 (2.94)
Average $Amp_{\mathrm{PR}}$ (o) (imp%)					
S1	11.13	13.28 (19.39)	11.67 (4.89)	13.51 (21.46)	11.71 (5.21)
S2	9.56	10.16 (6.25)	10.62 (11.08)	13.72 (43.47)	13.59 (42.13)
S3	13.28	13.47 (1.39)	17.54 (32.07)	19.00 (43.06)	16.87 (27.00)
S4	6.74	5.41 (−19.83)	7.92 (17.43)	9.24 (37.08)	8.89 (31.80)
S5	10.51	11.67 (11.11)	10.19 (−3.00)	9.53 (−9.31)	9.05 (−13.91)
S6	12.51	12.81 (2.38)	17.66 (41.11)	22.23 (77.66)	22.86 (82.66)
S7	12.56	23.42 (86.53)	25.08 (99.71)	24.73 (96.94)	35.14 (179.85)
S8	7.97	7.53 (−5.51)	10.47 (31.45)	11.19 (40.51)	10.13 (27.14)
S9	10.99	16.76 (52.44)	16.11 (46.53)	14.68 (33.52)	11.38 (3.50)
S10	14.94	14.44 (−3.31)	14.96 (0.16)	15.40 (3.07)	14.94 (0.02)
Average walking speed (rpm) (imp%)					
S1	450.22	476.31 (5.79)	476.31 (5.79)	476.31 (5.79)	471.68 (4.76)
S2	179.83	255.36 (42.00)	259.28 (44.18)	273.70 (52.20)	243.90 (35.63)
S3	95.77	225.76 (135.74)	275.21 (187.37)	384.43 (301.42)	476.31 (397.37)
S4	451.69	476.31 (5.45)	476.31 (5.45)	476.31 (5.45)	476.31 (5.45)
S5	319.82	476.31 (48.93)	476.31 (48.93)	476.31 (48.93)	474.44 (48.35)
S6	158.07	213.32 (34.95)	183.42 (16.03)	251.24 (58.94)	190.42 (20.46)
S7	185.19	315.25 (70.23)	326.21 (76.15)	336.05 (81.46)	324.22 (75.07)
S8	139.26	254.91 (83.04)	258.14 (85.36)	346.88 (149.08)	350.97 (152.02)
S9	177.18	332.32 (87.56)	313.66 (77.03)	299.05 (68.78)	308.09 (73.88)
S10	302.76	474.58 (56.75)	475.92 (57.19)	476.31 (57.32)	466.09 (53.95)
Average stride length (cm) (imp%)					
S1	68.93	67.97 (−1.40)	69.31 (0.54)	71.65 (3.95)	72.17 (4.70)
S2	46.36	46.42 (0.13)	47.32 (2.06)	48.41 (4.42)	49.92 (7.68)
S3	27.12	44.15 (62.83)	38.76 (42.95)	51.50 (89.93)	70.04 (158.31)
S4	63.97	64.51 (0.84)	65.45 (2.31)	64.46 (0.76)	67.20 (5.05)
S5	48.17	53.23 (10.50)	56.52 (17.33)	54.88 (13.93)	56.24 (16.75)
S6	48.45	39.53 (−18.4)	60.67 (25.23)	44.55 (−8.05)	40.62 (−16.15)
S7	31.33	54.14 (72.83)	76.00 (142.62)	50.96 (62.68)	50.90 (62.47)
S8	48.61	57.11 (17.48)	62.90 (29.4)	55.04 (13.22)	55.48 (14.13)
S9	59.65	56.63 (−5.07)	66.88 (12.12)	53.44 (−10.42)	54.59 (−8.48)
S10	54.34	64.03 (17.82)	67.69 (24.57)	67.23 (23.71)	65.38 (20.31)

imp%: percentage improvement compared with Stage *A*.

### 3.2 Animated stepping cue

The performance indexes from experiments using animated stepping cues are shown in Supplementary Figures S3–S4 and Supplementary Tables S6–S7 in the Supplementary Material. Comparable overall improvements were observed following NDT training, including enhanced longitudinal symmetry, increased pelvic rotation, and gains in both walking speed and stride length.

The healthy participants’ performance indexes with animated stepping cues are given in Table 3. All healthy participants’ longitudinal symmetry exhibited improvement at all stages; six participants reached the highest improvement at stage *D* and three reached it at stage $\bar{B}$ . The highest average improvement was 34.23% at stage *D*, showing the immediate and sustained effects of visual cues in improving $Asym_{SP}$ . Eight participants showed improvements in pelvic rotation across all stages (the exceptions were H2 and H5). At stage $\bar{A}$ , five participants achieved the largest improvement, with the best average improvement of 30.56%. These findings suggest delayed effects of animated stepping cues in enhancing ${Amp}_{\mathrm{PR}}$ . Furthermore, similar improvements in walking speed were observed for all participants at every stage. All participants achieved the maximum treadmill speed at stage *D*, with an average improvement of 95.64%. All participants exhibited improvements in stride length across all stages, with a highest average improvement of 31.55% at stage $\bar{B}$ , suggesting sustained effects of animated stepping cues.

**TABLE 3:** Performance indexes and improvements with animated stepping cues (healthy subjects).

Subject	A	B	D	$\bar{B}$	$\bar{A}$
Average $Asym_{SP}$ (%) (imp%)					
H1	15.54	12.25 (21.18)	10.56 (32.04)	7.18 (53.76)	11.61 (25.27)
H2	23.30	12.09 (48.11)	11.81 (49.29)	11.46 (50.81)	14.99 (35.66)
H3	9.98	9.14 (8.35)	8.60 (13.74)	9.65 (3.28)	9.74 (2.37)
H4	22.82	18.18 (20.32)	17.24 (24.45)	16.27 (28.70)	19.84 (13.05)
H5	31.07	29.56 (4.87)	13.55 (56.41)	20.62 (33.65)	16.95 (45.44)
H6	13.98	12.44 (11.03)	11.58 (17.20)	12.07 (13.70)	12.97 (7.20)
H7	16.79	13.46 (19.87)	7.85 (53.25)	8.10 (51.79)	9.86 (41.29)
H8	14.67	9.14 (37.73)	7.65 (47.83)	8.90 (39.34)	9.48 (35.41)
H9	19.43	11.99 (38.33)	11.56 (40.49)	12.94 (33.44)	15.16 (21.99)
H10	11.58	8.95 (22.70)	10.71 (7.54)	9.93 (14.22)	9.72 (16.06)
Average $Amp_{\mathrm{PR}}$ (o) (imp%)					
H1	8.41	8.65 (2.84)	13.39 (59.24)	13.09 (55.64)	13.86 (64.86)
H2	15.61	12.68 (−18.81)	10.42 (−33.26)	9.68 (−38.02)	12.53 (−19.76)
H3	3.34	3.34 (0.12)	4.31 (29.19)	4.78 (43.04)	5.38 (61.27)
H4	10.87	10.25 (−5.74)	11.23 (3.31)	11.93 (9.77)	12.58 (15.71)
H5	19.13	17.83 (-6.77)	8.11 (−57.60)	12.32 (-35.59)	15.31 (−19.94)
H6	9.77	13.45 (37.69)	23.02 (135.63)	17.60 (80.19)	12.93 (32.34)
H7	5.37	5.45 (1.55)	5.84 (8.71)	6.91 (28.80)	7.62 (41.99)
H8	10.82	15.12 (39.70)	13.27 (22.63)	13.07 (20.73)	12.94 (19.52)
H9	6.67	9.54 (42.97)	9.71 (45.52)	10.22 (53.12)	12.83 (92.27)
H10	6.45	9.05 (40.25)	7.17 (11.09)	7.81 (20.99)	7.58 (17.51)
Average walking speed (rpm) (imp%)					
H1	335.99	446.63 (32.93)	476.31 (41.76)	476.31 (41.76)	476.31 (41.76)
H2	183.58	468.60 (155.26)	476.31 (159.46)	476.31 (159.46)	463.18 (152.31)
H3	331.69	464.38 (40.00)	476.31 (43.60)	476.31 (43.60)	476.31 (43.60)
H4	179.03	457.22 (155.39)	476.31 (166.05)	476.31 (166.05)	464.60 (159.51)
H5	315.96	385.35 (21.96)	476.31 (50.75)	476.31 (50.75)	476.31 (50.75)
H6	157.02	334.70 (113.16)	476.31 (203.35)	476.31 (203.35)	476.31 (203.35)
H7	235.97	356.81 (51.21)	476.31 (101.85)	476.31 (101.85)	476.31 (101.85)
H8	313.12	470.84 (50.37)	475.99 (52.02)	439.54 (40.38)	476.31 (52.12)
H9	325.83	458.55 (40.73)	476.31 (46.18)	476.31 (46.18)	476.31 (46.18)
H10	249.01	476.04 (91.17)	476.31 (91.28)	476.31 (91.28)	476.31 (91.28)
Average stride length (cm) (imp%)					
H1	81.54	103.56 (27.01)	91.20 (11.85)	89.36 (9.59)	87.96 (7.87)
H2	52.53	83.70 (59.34)	79.50 (51.35)	87.04 (65.71)	86.53 (64.74)
H3	54.12	66.71 (23.26)	69.24 (27.93)	69.59 (28.58)	64.70 (19.54)
H4	55.10	86.98 (57.86)	91.11 (65.35)	87.60 (58.98)	91.73 (66.48)
H5	79.24	88.68 (11.91)	80.32 (1.36)	84.79 (7.00)	83.65 (5.57)
H6	64.60	76.51 (18.44)	86.21 (33.44)	81.18 (25.67)	83.57 (29.37)
H7	43.85	49.37 (12.58)	65.64 (49.69)	67.58 (54.10)	65.16 (48.59)
H8	68.22	85.60 (25.47)	89.86 (31.72)	83.97 (23.08)	79.75 (16.90)
H9	56.57	69.04 (22.05)	69.13 (22.21)	70.23 (24.16)	70.76 (25.09)
H10	59.29	74.85 (26.24)	68.28 (15.15)	70.31 (18.58)	71.64 (20.83)

imp%: percentage improvement compared with Stage *A*.

After confirming the safety and promising rehabilitative effects of this intervention in healthy individuals, clinical experiments with animated cues were extended to ten post-stroke patients. The results are illustrated in Table 4. Nearly all patients showed improved longitudinal symmetry at all stages, with one exception (S16) at stage *D*. At stage $\bar{A}$ , five patients achieved their greatest improvement, with an average improvement of 22.42%, demonstrating the delayed impacts of visual cues on $Asym_{SP}$ . Nine patients exhibited increased pelvic rotation at stages *D* and $\bar{B}$ (the exception was S12). At stage $\bar{B}$ , four patients achieved their best improvement, and the average improvement was 31.34%, indicating sustained effects of animated cues on pelvic rotation. Similarly, patients demonstrated walking speeds similar to healthy subjects at stage *A*, but slower speeds after the intervention, which may be attributable to underlying physical constraints. Walking speed improved for all stroke participants, with seven subjects achieving their highest gains at stage $\bar{B}$ . The highest average improvement was 138.05% at stage $\bar{B}$ , again showing the delayed effects of animated stepping cues. Almost all patients showed increased stride length across all stages (the exceptions were S11 at Stage *B* and S19 at Stage $\bar{B}$ ). Among the patients, five had their highest improvements at stage $\bar{B}$ , while the largest average improvement was 55.17% at stage $\bar{A}$ . These findings confirm that integrating animated stepping cues and tactile stimulation can enhance gait performance in stroke rehabilitation, but the effects are delayed.

**TABLE 4:** Performance indexes and improvements with animated stepping cues (stroke patients).

​	A	B	D	$\bar{B}$	$\bar{A}$
Average $Asym_{SP}$ (%) (imp%)					
S11	21.26	21.23 (0.15)	20.42 (3.97)	19.24 (9.48)	20.78 (2.27)
S12	19.52	12.90 (33.93)	14.40 (26.24)	15.61 (20.02)	18.21 (6.72)
S13	21.08	15.60 (26.00)	15.40 (26.98)	17.60 (16.54)	17.78 (15.67)
S14	22.19	18.62 (16.10)	16.61 (25.12)	18.79 (15.33)	16.57 (25.30)
S15	15.85	9.26 (41.60)	8.59 (45.84)	9.26 (41.61)	8.46 (46.64)
S16	31.26	18.97 (39.32)	33.34 (−6.64)	19.91 (36.33)	18.28 (41.54)
S17	35.92	35.06 (2.38)	30.45 (15.24)	30.69 (14.56)	30.05 (16.34)
S18	27.34	25.88 (5.34)	25.00 (8.57)	27.28 (0.22)	26.47 (3.18)
S19	13.13	9.60 (26.93)	8.33 (36.57)	8.81 (32.93)	7.30 (44.39)
S20	39.75	35.76 (10.04)	32.42 (18.43)	30.68 (22.82)	30.94 (22.17)
Average $Amp_{\mathrm{PR}}$ (o) (imp%)					
S11	9.76	14.32 (46.71)	14.56 (49.14)	12.41 (27.14)	12.25 (25.54)
S12	7.16	5.59 (−21.89)	5.41 (−24.40)	4.84 (−32.40)	6.02 (−15.84)
S13	10.09	12.45 (23.40)	14.56 (44.38)	15.92 (57.81)	17.41 (72.62)
S14	6.26	6.30 (0.65)	6.62 (5.74)	7.11 (13.64)	6.69 (6.94)
S15	13.94	12.57 (−9.85)	21.06 (51.09)	17.62 (26.38)	14.98 (7.42)
S16	12.43	15.04 (21.00)	12.97 (4.39)	13.01 (4.68)	12.94 (4.15)
S17	11.37	14.84 (30.49)	17.41 (53.04)	18.11 (59.23)	13.53 (18.93)
S18	4.73	5.27 (11.33)	6.77 (43.21)	9.92 (109.74)	10.02 (111.73)
S19	8.34	9.44 (13.20)	11.46 (37.47)	11.57 (38.73)	10.48 (25.65)
S20	16.17	15.88 (−1.80)	16.80 (3.88)	17.55 (8.48)	15.65 (−3.24)
Average walking speed (rpm) (imp%)					
S11	419.91	476.31 (13.43)	476.31 (13.43)	476.31 (13.43)	475.73 (13.29)
S12	177.64	425.84 (139.72)	476.31 (168.13)	476.31 (168.13)	401.07 (125.77)
S13	259.71	467.07 (79.84)	476.31 (83.40)	476.31 (83.40)	416.38 (60.33)
S14	138.10	319.22 (131.15)	382.42 (176.92)	475.66 (244.44)	316.50 (129.18)
S15	97.06	362.05 (273.03)	381.11 (292.67)	381.11 (292.67)	381.10 (292.66)
S16	141.41	215.29 (52.24)	221.40 (56.56)	222.25 (57.17)	222.31 (57.21)
S17	203.71	360.00 (76.72)	429.41 (110.79)	476.31 (133.82)	460.64 (126.12)
S18	131.87	334.54 (153.70)	476.31 (261.21)	476.31 (261.21)	476.31 (261.21)
S19	443.47	476.31 (7.41)	476.31 (7.41)	476.31 (7.41)	476.31 (7.41)
S20	129.73	212.13 (63.51)	254.35 (96.05)	283.95 (118.87)	249.54 (92.35)
Average stride length (cm) (imp%)					
S11	71.25	70.62 (−0.88)	76.52 (7.40)	75.50 (5.97)	95.10 (33.48)
S12	51.05	65.12 (27.55)	72.27 (41.56)	70.78 (38.64)	66.26 (29.78)
S13	67.51	80.49 (19.24)	76.19 (12.86)	75.98 (12.55)	68.83 (1.96)
S14	44.77	64.09 (43.14)	70.05 (56.47)	81.48 (82.00)	60.06 (34.15)
S15	41.50	78.10 (88.20)	67.90 (63.62)	69.16 (66.66)	94.77 (128.38)
S16	31.95	38.17 (19.45)	38.70 (21.11)	40.75 (27.53)	40.54 (26.89)
S17	32.54	49.77 (52.93)	56.18 (72.63)	60.36 (85.49)	59.49 (82.81)
S18	34.44	55.70 (61.73)	72.74 (111.21)	75.00 (111.76)	73.14 (112.35)
S19	59.81	60.64 (1.39)	61.37 (2.61)	58.26 (−2.59)	61.40 (2.65)
S20	28.19	42.22 (49.80)	51.73 (88.54)	58.42 (107.27)	56.16 (99.24)

imp%: percentage improvement compared with Stage *A*.

## 4 Discussion

This study presents an automated NDT trainer that integrates a visual stimulation system to deliver real-time cues to the contralateral ASIS upon detection of HSs. As shown in Tables 1–4, substantial inter-individual variability was observed across all measured parameters. In healthy participants at Stage *A*, swing-phase asymmetry ranged from 9.98% to 37.98%, pelvic rotation from 3.34° to 14.92°, walking speed from 108 to 419 rpm, and stride length from 38.54 to 87.86 cm at Stage *A*. Similar wide ranges were evident among post-stroke patients, with swing-phase asymmetry between 10.82% and 40.46%, pelvic rotation between 4.73° and 16.17°, walking speed between 97.06 and 451.69 rpm, and stride length from 27.12 to 71.25 cm. Given this pronounced variability, a within-subject analytical approach is more appropriate than cross-group comparisons for evaluating intervention effects.

The results from Tables 1–4 are synthesized in Table 5, where the most favorable outcomes are highlighted in bold. When using the flashing square cues, most healthy participants demonstrated their greatest improvements at Stage *D*. In contrast, post-stroke participants exhibited optimal outcomes across different metrics at different stages: longitudinal symmetry at Stage *D*, pelvic rotation at Stage *B̄*, walking speed at Stage *Ā*, and stride length at Stage *D*. With the animated stepping cues, healthy participants achieved their best performance in longitudinal symmetry, pelvic rotation, walking speed, and stride length at Stages *D*, *B̄*, *D*, and *Ā*, respectively. Post-stroke patients showed the most pronounced benefits from the animated stepping cues primarily at Stages *B̄* and *Ā*.

**TABLE 5:** Comparisons of Tables 1–4.

$N\;\left(\bar{\text{imp}\%}\right)$	B	D	$\bar{B}$	$\bar{A}$
	Healthy subjects with flashing square cues			
$Asym_{SP}$	0 (19.92)	**6** (**37.72**)	3 (34.63)	1 (28.39)
$Amp_{\mathrm{PR}}$	1 (8.64)	**4** (**23.93**)	0 (18.95)	**4** (23.13)
*Walking speed*	1 (144.86)	**10** (**173.41**)	**10** (**173.41**)	9 (170.88)
*Stride length*	2 (**37.84**)	**4** (37.44)	0 (34.55)	**4** (37.24)
Post-stroke patients with flashing square cues				
$Asym_{SP}$	0 (19.18)	**6** (**31.32**)	1 (25.41)	3 (22.56)
$Amp_{\mathrm{PR}}$	2 (15.08)	0 (28.14)	**6** (**38.74**)	2 (38.56)
*Walking speed*	4 (57.04)	3 (60.35)	**7** (82.94)	3 (**86.69**)
*Stride length*	0 (15.75)	**6** (**29.91**)	0 (19.41)	4 (26.47)
Healthy subjects with animated stepping cues				
$Asym_{SP}$	1 (23.24)	**6** (**34.23**)	3 (32.27)	0 (24.38)
$Amp_{\mathrm{PR}}$	2 (13.38)	1 (22.45)	0 (23.89)	**5** (**30.56**)
*Walking speed*	0 (75.22)	**10** (**95.64**)	9 (94.47)	8 (94.27)
*Stride length*	**3** (28.42)	2 (31.01)	**3** (**31.55**)	2 (30.50)
Post-stroke patients with animated stepping cues				
$Asym_{SP}$	1 (20.17)	2 (20.02)	2 (20.97)	**5** (**22.42**)
$Amp_{\mathrm{PR}}$	1 (11.33)	2 (26.78)	**4** (**31.34**)	2 (25.39)
*Walking speed*	2 (99.07)	6 (126.66)	**7** (**138.05**)	3 (116.55)
*Stride length*	1 (36.26)	1 (47.30)	**5** (54.13)	3 (**55.17**)

N: number of subjects with the highest improvements at the specified stage.

$\bar{\text{imp}\%}$ **:** average improvements of the group at the specified stage.

Bold represents the most favorable outcomes.

From Table 5, the post-stroke group demonstrated more pronounced improvements at later stages than the healthy group. This pattern suggests that stroke survivors may rely more heavily on carryover effects due to impairments in intrinsic timing mechanisms, thereby exhibiting stronger short-term motor memory responses. Additionally, participants required more time to process the animated stepping cues than the flashing square cues. One explanation is that the flashing cues provide simple, low-load visual feedback that supports rapid online correction, whereas the animated cues impose greater cognitive and attentional demands and may require more time for mapping observed actions onto self-generated gait adjustments.

Importantly, the system provides multimodal feedback, utilizing the neuroanatomical independence of sensory processing. Previous research has demonstrated that visuomotor and kinesthetic learning differ neuroanatomically and behaviorally: visuomotor learning engages cerebellar and parietal circuits using visual information, whereas kinesthetic learning relies on proprioceptive and tactile feedback and engages somatosensory cortices (Rabe et al., 2009; Donchin et al., 2012). This independence suggests that stroke survivors with deficits in utilizing one specific feedback modality may still effectively process and adapt to alternative sensory inputs (Moore and Cluff, 2021). Our system integrates tactile guidance with synchronized visual stimulation to ensure that motor modulation is facilitated even when single sensory channels are compromised.

Overall, the rehabilitative effects were stronger for the flashing square cues than for the animated stepping cues. This superior performance may be attributed to the visually salient and straightforward design of the flashing square cues, which facilitates gait adjustment by enabling participants to observe and respond to their own gait feedback. Conversely, while the animated stepping cues emphasize lower-limb movement, their configuration may require greater attentional resources for processing, thereby potentially reducing their effectiveness in post-stroke patients. Nevertheless, the animated stepping cues align with AOT principles by embedding the walking task within a goal-oriented context. This task-driven design appears to enhance participant engagement and motivation and contributed notably to improvements in walking speed. The system employs error-based feedback as a clinical rationale to facilitate gait adjustment. While visuomotor adaptation can be impaired in many individuals after stroke (Moore et al., 2022; 2024; 2025; Scheidt and Stoeckmann, 2007; Mutha et al., 2011), providing external guidance may assist those in Brunnstrom Stages III–V to achieve immediate motor modulation, despite inherent heterogeneity in their learning capacity.

It is important to acknowledge that this study is preliminary, with a relatively small sample size and a heterogeneous post-stroke cohort. Variability in stroke severity, sensory impairment, and capacity to respond to cues may affect responsiveness to visual and tactile cues, and thus, the current findings should be regarded as preliminary evidence that requires further validation. Another limitation is the absence of a delayed retention test. Consequently, the observed improvements may primarily reflect transient performance gains and the initial encoding phase of motor memory, rather than long-term motor consolidation. Future longitudinal studies incorporating retention assessments and evaluations of real-world generalization are essential to confirm the durability of gait training effects and the clinical utility of the visual-tactile cueing system.

## 5 Conclusion

This paper introduces a visual stimulation system and its integration with an NDT trainer for the delivery of multisensory interventions aimed at enhancing gait rehabilitation in post-stroke patients. Two interactive visual training interfaces were developed: flashing square cues based on the VFT principles and animated stepping cues based on the AOT principles. The visual stimulation system was then integrated with the NDT trainer to provide both tactile and visual cues for clinical experiments involving healthy individuals and post-stroke patients.

The experimental results demonstrate that the proposed visual stimulation system has the potential to enhance rehabilitation outcomes. Among the two interfaces, the flashing square cues produced the most pronounced improvements in gait performance, indicating that simple, intuitive visual cues can effectively facilitate gait modulation and motor learning. These findings suggest that visual cueing, when appropriately designed, can serve as an effective adjunct to conventional NDT-based rehabilitation.

Despite these promising results, this study is limited by the relatively small sample size. Future work should involve larger participant cohorts and more robust statistical analyses to further validate the effectiveness of the proposed approach. Building on the observed benefits of integrating NDT principles with visual cueing strategies, future developments will focus on incorporating additional sensory modalities, such as auditory cues, to further promote natural gait patterns and support motor relearning through enhanced sensory–motor integration.

## Data Availability

The raw data supporting the conclusions of this article will be made available by the authors, without undue reservation.
