# Supplementary material for: Visual stimulation in automated gait rehabilitation for post-stroke patients
Source: Front Bioeng Biotechnol. 2026 May 28;14:1769011. doi: 10.3389/fbioe.2026.1769011 (PMC13253541; doi:10.3389/fbioe.2026.1769011)
Supplement: Supplementary file 1 [file Supplementaryfile1.docx]

Supplementary Material

Table S1. System specifications.

| Motion Capture System | |
| --- | --- |
| IMU | |
| dimensions | $43.7\times39.7\times13.7\text{ mm}$ |
| sampling rate | <128 Hz |
| gyroscope | ± 2000 deg/s |
| accelerometer | ± 200 g |
| magnetometer | ± 8 Gauss |
| Cable Control System | |
| Step Motor Driver | |
| pulse rate | <500 kHz |
| Weight | 975 g |
| Size | 175 x 119 x 45 mm |
| Step Motor | |
| voltage | 1.75 V |
| torque | 16 kgf.cm |
| weight | 1.2 kg |
| length | 84 mm |
| Load Cell | |
| force | 0-200 lb |
| voltage | 0~10 V |
| Treadmill Control System | |
| Infrared Rangefinder | |
| input voltage | 0~5 V |
| measured distance | 10~80 cm |
| weight | 3.5 g |
| DC Brushless Motor Driver | |
| operating voltage | 24 VDC |
| input signal | 50~50k Hz (PWM/PFM) |
| maximum output current | 45 A |
| DC Brushless Motor | |
| power | 350 W |
| rated torque | 1.1 Nm |
| reduction ratio | 5 |
| Visual Assistance System | |
| Micro Controller | |
| CPU | ATmega 328P |
| operating voltage | 5 V |
| interface | UART、$I^{2}C$、SPI |
| Monitor | |
| operating voltage | 100V~240V AC |
| resolution | 1920x1080 Full HD |
| size | 22 inch |

Table S2. Demographic data of the healthy subjects.

| Subject | Gender | Age | Height  (cm) | Weight  (kg) |
| --- | --- | --- | --- | --- |
| H1 | Male | 29 | 173 | 68 |
| H2 | Male | 30 | 184 | 100 |
| H3 | Male | 27 | 171 | 60 |
| H4 | Male | 31 | 170 | 62 |
| H5 | Female | 25 | 161 | 58 |
| H6 | Male | 24 | 176 | 80 |
| H7 | Female | 20 | 165 | 60 |
| H8 | Male | 18 | 171 | 55 |
| H9 | Male | 23 | 175 | 68 |
| H10 | Male | 24 | 167 | 59 |

Table S3. Demographic data of the post-stroke patients for experiments with flashing square cues.

| Subject | Gender | Age | Height  (cm) | Weight  (kg) | Paretic  Side | BS  (Stage) | Type | Years | Area |
| --- | --- | --- | --- | --- | --- | --- | --- | --- | --- |
| S1 | Male | 45 | 170 | 65 | Right | IV | Hemorrhage | 4 | Basal ganglion |
| S2 | Female | 66 | 161 | 54 | Right | V | Hemorrhage | 7 | Basal ganglion |
| S3 | Male | 42 | 173 | 79 | Right | IV | Hemorrhage | 10 | Basal ganglion |
| S4 | Female | 55 | 154 | 60 | Left | IV | Ischemic | 2 | Thalumus |
| S5 | Female | 57 | 158 | 77 | Right | IV | Ischemic | 7 | ACA,MCA |
| S6 | Female | 53 | 165 | 68 | Right | IV | Hemorrhage | 4 | Basal ganglion |
| S7 | Male | 58 | 170 | 77 | Right | V | Ischemic | 2 | Corona radiata |
| S8 | Male | 47 | 172 | 72 | Left | IV | Hemorrhage | 10 | Basal ganglion |
| S9 | Female | 52 | 155 | 57 | Right | V | Hemorrhage | 3 | Basal ganglion |
| S10 | Male | 63 | 169 | 65 | Left | IV | Hemorrhage | 13 | Basal ganglion |

Table S4. Demographic data of the post-stroke patients for experiments with animated stepping cues.

| Subject | Gender | Age | Height  (cm) | Weight  (kg) | Paretic  Side | BS  (Stage) | Type | Years | Area |
| --- | --- | --- | --- | --- | --- | --- | --- | --- | --- |
| S11 | Male | 46 | 170 | 65 | Right | V | Hemorrhage | 4 | Basal ganglion |
| S12 | Female | 65 | 170 | 75 | Right | V | Ischemic | 7 | MCA |
| S13 | Male | 66 | 165 | 64 | Right | IV | Ischemic | 3 | Corona radiata |
| S14 | Male | 60 | 173 | 73 | Right | III | Ischemic | 12 | Thalamus |
| S15 | Male | 42 | 173 | 75 | Right | IV | Hemorrhage | 0.5 | Basal ganglion |
| S16 | Male | 49 | 173 | 75 | Right | III | Hemorrhage | 2 | Basal ganglion |
| S17 | Male | 53 | 169 | 67 | Right | III | Hemorrhage | 14 | Basal ganglion |
| S18 | Male | 47 | 172 | 72 | Left | V | Hemorrhage | 10 | Basal ganglion |
| S19 | Male | 42 | 173 | 79 | Right | V | Hemorrhage | 10 | Basal ganglion |
| S20 | Female | 64 | 165 | 66 | Right | III | Hemorrhage | 5 | Basal ganglion |

|   (a) Asymmetry of Swing Phase |   (b) Pelvic Rotation | (c) Average Speed |   (d) Average Left Stride Length |   (e) Average Right Stride Length |
| --- | --- | --- | --- | --- |
| 1. Subject H1. | | | | |
|   (a) Asymmetry of Swing Phase |   (b) Pelvic Rotation |  (c) Average Speed |   (d) Average Left Stride Length |   (e) Average Right Stride Length |
| 1. Subject H2. | | | | |
|   (a) Asymmetry of Swing Phase |   (b) Pelvic Rotation |  (c) Average Speed |   (d) Average Left Stride Length |   (e) Average Right Stride Length |
| 1. Subject H3. | | | | |
|   (a) Asymmetry of Swing Phase |   (b) Pelvic Rotation |  (c) Average Speed |   (d) Average Left Stride Length |   (e) Average Right Stride Length |
| 1. Subject H4. | | | | |
|   (a) Asymmetry of Swing Phase |   (b) Pelvic Rotation |  (c) Average Speed |   (d) Average Left Stride Length |   (e) Average Right Stride Length |
| 1. Subject H5. | | | | |
|   (a) Asymmetry of Swing Phase |   (b) Pelvic Rotation |  (c) Average Speed |   (d) Average Left Stride Length |   (e) Average Right Stride Length |
| 1. Subject H6. | | | | |
|   (a) Asymmetry of Swing Phase |   (b) Pelvic Rotation |  (c) Average Speed |   (d) Average Left Stride Length |   (e) Average Right Stride Length |
| 1. Subject H7. | | | | |
|   (a) Asymmetry of Swing Phase |   (b) Pelvic Rotation |  (c) Average Speed |   (d) Average Left Stride Length |   (e) Average Right Stride Length |
| 1. Subject H8. | | | | |
|   (a) Asymmetry of Swing Phase |   (b) Pelvic Rotation |  (c) Average Speed |   (d) Average Left Stride Length |   (e) Average Right Stride Length |
| 1. Subject H9. | | | | |
|   (a) Asymmetry of Swing Phase |   (b) Pelvic Rotation |  (c) Average Speed |   (d) Average Left Stride Length |   (e) Average Right Stride Length |
| 1. Subject H10. | | | | |

Figure S1. Experiments with flashing square cues (healthy subjects).

Table S4. Performance indexes with flashing square cues (healthy subjects)

|  | | *A* | | *B* | | | | *D* | | | | $\bar{B}$ | | | $\bar{A}$ | | |  |
| --- | --- | --- | --- | --- | --- | --- | --- | --- | --- | --- | --- | --- | --- | --- | --- | --- | --- | --- |
| Asymmetry of the Swing Phase (%) (average ± standard derivation) | | | | | | | | | | | | | | | | | |  |
| H1 | 22.34±8.58 | | 13.67 ± 7.59 | | | 11.61 ± 7.82 | | | | 11.61 ± 7.55 | | | 14.53 ± 9.43 | | |  |  |  |
| H2 | 15.92±12.85 | | 15.00 ± 14.21 | | | 10.00 ± 7.40 | | | | 13.94 ± 9.36 | | | 15.88 ± 16.01 | | |  |  |  |
| H3 | 11.88 ± 8.92 | | 11.74 ± 10.10 | | | 10.52 ± 19.37 | | | | 9.67 ± 6.42 | | | 11.63 ± 7.73 | | |  |  |  |
| H4 | 21.54 ± 9.93 | | 17.54 ± 9.66 | | | 16.92 ± 5.48 | | | | 16.96 ± 6.89 | | | 17.42 ± 7.75 | | |  |  |  |
| H5 | 37.98±13.07 | | 23.94 ± 8.05 | | | 13.88 ± 6.93 | | | | 15.97 ± 6.92 | | | 20.05 ± 6.45 | | |  |  |  |
| H6 | 10.64±11.56 | | 7.11 ± 5.31 | | | 5.79 ± 4.37 | | | | 5.60 ± 4.12 | | | 5.80 ± 4.50 | | |  |  |  |
| H7 | 16.85±12.35 | | 13.73 ± 16.09 | | | 7.76 ± 6.01 | | | | 8.21 ± 6.45 | | | 7.73 ± 5.23 | | |  |  |  |
| H8 | 13.88±8.98 | | 13.63 ± 7.46 | | | 11.81 ± 17.59 | | | | 10.86 ± 7.81 | | | 11.28 ± 6.13 | | |  |  |  |
| H9 | 13.62±11.57 | | 10.16 ± 8.56 | | | 7.08 ± 5.88 | | | | 7.72 ± 5.54 | | | 9.63 ± 6.45 | | |  |  |  |
| H10 | 20.63±7.16 | | 16.71 ± 6.16 | | | 13.78 ± 6.77 | | | | 15.62 ± 7.23 | | | 13.90 ± 6.66 | | |  |  |  |
| Amplitude of Pelvic Rotation (^o^) (average ± standard derivation) | | | | | | | | | | | | | | | | | |  |
| H1 | 8.01 ± 1.49 | | 9.51 ± 4.72 | | | | 16.83 ± 3.99 | | | | 13.94 ± 3.19 | | | 16.63 ± 3.79 | | |  |  |
| H2 | 14.81 ± 4.18 | | 14.37 ± 5.01 | | | | 8.22 ± 2.61 | | | | 7.15 ± 1.93 | | | 7.21 ± 3.23 | | |  |  |
| H3 | 8.15 ± 1.76 | | 7.14 ± 1.87 | | | | 8.97 ± 2.14 | | | | 7.17 ± 1.93 | | | 4.84 ± 2.01 | | |  |  |
| H4 | 8.28 ± 1.52 | | 11.85 ± 2.80 | | | | 15.57 ± 2.32 | | | | 12.61 ± 2.30 | | | 9.09 ± 1.60 | | |  |  |
| H5 | 14.92 ± 2.92 | | 12.15 ± 2.49 | | | | 17.76 ± 3.14 | | | | 22.43 ± 3.64 | | | 23.75 ± 6.23 | | |  |  |
| H6 | 9.52 ± 2.10 | | 8.48 ± 2.68 | | | | 10.16 ± 3.26 | | | | 12.21 ± 2.64 | | | 14.95 ± 3.64 | | |  |  |
| H7 | 5.17 ± 2.35 | | 6.17 ± 1.98 | | | | 6.31 ± 2.84 | | | | 6.67 ± 2.67 | | | 7.36 ± 3.20 | | |  |  |
| H8 | 10.73 ± 1.39 | | 11.19 ± 2.03 | | | | 11.52 ± 2.14 | | | | 11.33 ± 2.76 | | | 11.42 ± 1.72 | | |  |  |
| H9 | 5.86 ± 2.10 | | 7.69 ± 2.66 | | | | 6.10 ± 1.74 | | | | 5.94 ± 1.81 | | | 6.40 ± 1.73 | | |  |  |
| H10 | 8.45 ± 2.43 | | 9.68 ± 1.38 | | | | 9.83 ± 1.85 | | | | 9.49 ± 1.51 | | | 11.12 ± 2.61 | | |  |  |
| Walking speed (rpm) (average ± standard derivation) | | | | | | | | | | | | | | | | | |  |
| H1 | 137.90±0.0039 | | 377.60 ± 0.0911 | | 476.31 ± 0.0012 | | | | 476.31 ± 0.0011 | | | | | 476.31 ± 0.0010 | | |  |  |
| H2 | 129.50±0.0546 | | 438.40 ± 0.0877 | | 476.31 ± 0.0018 | | | | 476.31 ± 0.0021 | | | | | 443.60 ± 0.0756 | | |  |  |
| H3 | 419.20±0.0601 | | 476.31 ± 0.0013 | | 476.31 ± 0.0010 | | | | 476.31 ± 0.0009 | | | | | 476.31 ± 0.0011 | | |  |  |
| H4 | 200.20±0.1024 | | 469.20 ± 0.0242 | | 476.31 ± 0.0009 | | | | 476.31 ± 0.0009 | | | | | 476.31 ± 0.0009 | | |  |  |
| H5 | 269.60±0.0464 | | 396.50 ± 0.0541 | | 476.31 ± 0.0010 | | | | 476.31 ± 0.0012 | | | | | 476.31 ± 0.0013 | | |  |  |
| H6 | 108.00±0.0153 | | 401.70 ± 0.1178 | | 476.31 ± 0.0011 | | | | 476.31 ± 0.0010 | | | | | 476.31 ± 0.0009 | | |  |  |
| H7 | 162.00±0.1062 | | 401.90 ± 0.0983 | | 476.31 ± 0.0008 | | | | 476.31 ± 0.0008 | | | | | 476.31 ± 0.0008 | | |  |  |
| H8 | 283.60±0.0206 | | 452.20 ± 0.0658 | | 476.31 ± 0.0011 | | | | 476.31 ± 0.0009 | | | | | 476.31 ± 0.0010 | | |  |  |
| H9 | 132.80±0.0836 | | 452.40 ± 0.0892 | | 476.31 ± 0.0012 | | | | 476.31 ± 0.0014 | | | | | 476.31 ± 0.0016 | | |  |  |
| H10 | 206.20±0.0727 | | 455.70 ± 0.0713 | | 476.31 ± 0.0014 | | | | 476.31 ± 0.0014 | | | | | 476.31 ± 0.0013 | | |  |  |
| Stride length (cm) (average ± standard derivation) | | | | | | | | | | | | | | | | | | |
| H1 | 87.86 ± 61.91 | | 83.95 ± 9.64 | | 101.25 ± 3.15 | | | | 99.95 ± 8.40 | | | | | 101.37 ± 2.66 | | |  |  |
| H2 | 57.26 ± 22.70 | | 108.33 ± 24.93 | | 68.91 ± 7.95 | | | | 61.50 ± 7.96 | | | | | 58.12 ± 12.53 | | |  |  |
| H3 | 68.67 ± 7.91 | | 69.65 ± 3.61 | | 81.38 ± 5.74 | | | | 77.24 ± 4.51 | | | | | 74.78 ± 3.85 | | |  |  |
| H4 | 55.56 ± 6.30 | | 90.17 ± 5.74 | | 97.08 ± 9.33 | | | | 95.86 ± 3.57 | | | | | 92.70 ± 3.11 | | |  |  |
| H5 | 65.56 ± 17.15 | | 75.49 ± 8.40 | | 77.92 ± 3.97 | | | | 85.49 ± 3.29 | | | | | 87.06 ± 3.27 | | |  |  |
| H6 | 58.40 ± 34.47 | | 83.19 ± 13.29 | | 78.36 ± 5.83 | | | | 86.31 ± 5.70 | | | | | 89.31 ± 3.90 | | |  |  |
| H7 | 38.54 ± 15.30 | | 58.61 ± 10.40 | | 64.88 ± 7.02 | | | | 65.01 ± 5.25 | | | | | 74.33 ± 3.65 | | |  |  |
| H8 | 70.93 ± 14.46 | | 85.13 ± 16.88 | | 89.70 ± 22.05 | | | | 77.03 ± 18.36 | | | | | 78.98 ± 2.12 | | |  |  |
| H9 | 39.64 ± 18.87 | | 62.20 ± 9.79 | | 65.06 ± 7.39 | | | | 62.23 ± 7.47 | | | | | 63.03 ± 2.95 | | |  |  |
| H10 | 48.81 ± 8.87 | | 69.94 ± 9.80 | | 65.20 ± 3.76 | | | | 61.91 ± 4.57 | | | | | 63.84 ± 2.68 | | |  |  |

|   (a) Asymmetry of Swing Phase |   (b) Pelvic Rotation |  (c) Average Speed |   (d) Average Left Stride Length |   (e) Average Right Stride Length |
| --- | --- | --- | --- | --- |
| 1. Subject S1. | | | | |
|   (a) Asymmetry of Swing Phase |   (b) Pelvic Rotation |  (c) Average Speed |   (d) Average Left Stride Length |   (e) Average Right Stride Length |
| 1. Subject S2. | | | | |
|   (a) Asymmetry of Swing Phase |   (b) Pelvic Rotation |  (c) Average Speed |   (d) Average Left Stride Length |   (e) Average Right Stride Length |
| 1. Subject S3. | | | | |
|   (a) Asymmetry of Swing Phase |   (b) Pelvic Rotation |  (c) Average Speed |   (d) Average Left Stride Length |   (e) Average Right Stride Length |
| 1. Subject S4. | | | | |
|   (a) Asymmetry of Swing Phase |   (b) Pelvic Rotation |  (c) Average Speed |   (d) Average Left Stride Length |   (e) Average Right Stride Length |
| 1. Subject S5. | | | | |
|   (a) Asymmetry of Swing Phase |   (b) Pelvic Rotation |  (c) Average Speed |   (d) Average Left Stride Length |   (e) Average Right Stride Length |
| 1. Subject S6. | | | | |
|   (a) Asymmetry of Swing Phase |   (b) Pelvic Rotation |  (c) Average Speed |   (d) Average Left Stride Length |   (e) Average Right Stride Length |
| 1. Subject S7. | | | | |
|   (a) Asymmetry of Swing Phase |   (b) Pelvic Rotation |  (c) Average Speed |   (d) Average Left Stride Length |   (e) Average Right Stride Length |
| 1. Subject S8. | | | | |
|   (a) Asymmetry of Swing Phase |   (b) Pelvic Rotation |  (c) Average Speed |   (d) Average Left Stride Length |   (e) Average Right Stride Length |
| 1. Subject S9. | | | | |
|   (a) Asymmetry of Swing Phase |   (b) Pelvic Rotation |  (c) Average Speed |   (d) Average Left Stride Length | (e) Average Right Stride Length |
| 1. Subject S10. | | | | |

Figure S2. Experiments with flashing square cues (post-stroke patients).

Table S5. Performance indexes with flashing square cues (stroke patients)

|  | | *A* | | *B* | | | | *D* | | | | $\bar{B}$ | | | $\bar{A}$ | | | |
| --- | --- | --- | --- | --- | --- | --- | --- | --- | --- | --- | --- | --- | --- | --- | --- | --- | --- | --- |
| Asymmetry of the Swing Phase (%) (average ± standard derivation) | | | | | | | | | | | | | | | | | | |
| S1 | 19.53±11.10 | | 16.50±9.38 | | | 15.83±9.25 | | | | 16.27±9.93 | | | 18.06±11.63 | | |  |  |  |
| S2 | 33.86±18.99 | | 24.18±22.17 | | | 23.64±11.24 | | | | 23.23±11.39 | | | 26.28±11.20 | | |  |  |  |
| S3 | 40.46±20.51 | | 28.25±17.03 | | | 26.44±16.77 | | | | 26.50±15.64 | | | 32.15±14.27 | | |  |  |  |
| S4 | 17.80±9.59 | | 11.83±8.97 | | | 9.32±6.20 | | | | 9.62±6.54 | | | 9.09±6.94 | | |  |  |  |
| S5 | 10.82±12.52 | | 10.80±7.48 | | | 9.68±7.60 | | | | 9.18±6.86 | | | 9.13±6.83 | | |  |  |  |
| S6 | 35.16±13.30 | | 26.30±9.22 | | | 17.08±9.12 | | | | 19.56±9.45 | | | 14.00±9.58 | | |  |  |  |
| S7 | 20.51±14.82 | | 17.16±11.01 | | | 9.70±6.10 | | | | 17.16±8.76 | | | 14.55±9.52 | | |  |  |  |
| S8 | 34.82±24.19 | | 26.46±40.08 | | | 24.16±18.14 | | | | 24.42±11.33 | | | 29.76±11.00 | | |  |  |  |
| S9 | 24.03±12.67 | | 20.92±10.80 | | | 17.41±9.47 | | | | 21.04±13.12 | | | 23.10±12.99 | | |  |  |  |
| S10 | 40.29±7.67 | | 38.15±9.75 | | | 36.68±7.49 | | | | 37.32±6.84 | | | 39.11±6.99 | | |  |  |  |
| Amplitude of Pelvic Rotation (^o^) (average ± standard derivation) | | | | | | | | | | | | | | | | | |  |
| S1 | 11.13±4.01 | | 13.28±1.16 | | | | 11.67±1.09 | | | | 13.51±1.28 | | | 11.71±1.88 | | |  |  |
| S2 | 9.56±3.59 | | 10.16±2.54 | | | | 10.62±2.68 | | | | 13.72±2.79 | | | 13.59±3.41 | | |  |  |
| S3 | 13.28±2.34 | | 13.47±2.89 | | | | 17.54±3.74 | | | | 19.00±2.84 | | | 16.87±4.19 | | |  |  |
| S4 | 6.74±1.85 | | 5.41±1.59 | | | | 7.92±1.86 | | | | 9.24±2.37 | | | 8.89±1.47 | | |  |  |
| S5 | 10.51±1.84 | | 11.67±1.59 | | | | 10.19±1.91 | | | | 9.53±1.56 | | | 9.05±1.38 | | |  |  |
| S6 | 12.51±2.80 | | 12.81±2.59 | | | | 17.66±3.20 | | | | 22.23±3.66 | | | 22.86±6.12 | | |  |  |
| S7 | 12.56±4.56 | | 23.42±5.43 | | | | 25.08±7.99 | | | | 24.73±5.00 | | | 35.14±9.71 | | |  |  |
| S8 | 7.97±3.44 | | 7.53±2.10 | | | | 10.47±1.78 | | | | 11.19±1.85 | | | 10.13±1.84 | | |  |  |
| S9 | 10.99±2.30 | | 16.76±1.85 | | | | 16.11±3.41 | | | | 14.68±2.46 | | | 11.38±1.60 | | |  |  |
| S10 | 14.94±1.21 | | 14.44±1.32 | | | | 14.96±1.33 | | | | 15.40±1.44 | | | 14.94±1.09 | | |  |  |
| Walking speed (rpm) (average ± standard derivation) | | | | | | | | | | | | | | | | | |  |
| S1 | 450.22±0.1554 | | 476.31±0.0014 | | 476.30±0.0014 | | | | 476.31±0.0015 | | | | | 471.68±0.0093 | | |  |  |
| S2 | 179.83±0.0411 | | 255.36±0.0528 | | 259.28±0.0431 | | | | 273.70±0.0519 | | | | | 243.90±0.0772 | | |  |  |
| S3 | 95.77±0.0242 | | 225.76±0.1195 | | 275.21±0.0212 | | | | 384.43±0.0670 | | | | | 476.32±0.0012 | | |  |  |
| S4 | 451.69±0.0508 | | 476.31±0.0017 | | 476.31±0.0016 | | | | 476.31±0.0018 | | | | | 476.32±0.0017 | | |  |  |
| S5 | 319.82±0.2418 | | 476.31±0.0014 | | 476.31±0.0015 | | | | 476.31±0.0015 | | | | | 474.44±0.0140 | | |  |  |
| S6 | 158.07±0.1175 | | 213.32±0.0578 | | 183.42±0.0714 | | | | 251.24±0.0224 | | | | | 190.42±0.0212 | | |  |  |
| S7 | 185.19±0.0412 | | 315.25±0.0253 | | 326.21±0.0211 | | | | 336.05±0.0009 | | | | | 324.22±0.0044 | | |  |  |
| S8 | 139.26±0.1288 | | 254.91±0.1332 | | 258.14±0.1668 | | | | 346.88±0.0367 | | | | | 350.97±0.0955 | | |  |  |
| S9 | 177.18±0.1261 | | 332.32±0.0374 | | 313.66±0.0415 | | | | 299.05±0.0464 | | | | | 308.09±0.0034 | | |  |  |
| S10 | 302.76±0.1731 | | 474.58±0.0095 | | 475.92±0.0023 | | | | 476.31±0.0017 | | | | | 466.09±0.1050 | | |  |  |
| Stride length (cm) (average ± standard derivation) | | | | | | | | | | | | | | | | | | |
| S1 | 68.93 ± 37.57 | | 67.97 ± 8.43 | | 69.31 ± 6.89 | | | | 71.65 ± 6.34 | | | | | 72.17 ± 7.34 | | |  |  |
| S2 | 46.36 ± 13.27 | | 46.42 ± 7.52 | | 47.32 ± 5.36 | | | | 48.40 ± 8.24 | | | | | 49.92 ± 18.53 | | |  |  |
| S3 | 27.12 ± 14.10 | | 44.15 ± 19.12 | | 38.76 ± 8.66 | | | | 51.50 ± 9.17 | | | | | 70.04 ± 9.93 | | |  |  |
| S4 | 63.97 ± 10.06 | | 64.51 ± 11.69 | | 65.45 ± 8.19 | | | | 64.46 ± 8.76 | | | | | 67.20 ± 4.60 | | |  |  |
| S5 | 48.17 ± 20.46 | | 53.23 ± 12.81 | | 56.52 ± 17.57 | | | | 54.88 ± 15.36 | | | | | 56.24 ± 16.39 | | |  |  |
| S6 | 48.45 ± 19.57 | | 39.53 ± 16.08 | | 60.67 ± 150.56 | | | | 44.55 ± 13.13 | | | | | 40.62 ± 17.26 | | |  |  |
| S7 | 31.33 ± 5.79 | | 54.14 ± 4.46 | | 76.00 ± 5.60 | | | | 50.96 ± 2.29 | | | | | 50.90 ± 2.98 | | |  |  |
| S8 | 48.61 ± 23.45 | | 57.11 ± 19.17 | | 62.90 ± 21.99 | | | | 55.04 ± 17.26 | | | | | 55.48 ± 15.06 | | |  |  |
| S9 | 59.65 ± 54.16 | | 56.63 ± 9.17 | | 66.88 ± 65.61 | | | | 53.44 ± 10.64 | | | | | 54.59 ± 2.38 | | |  |  |
| S10 | 54.34 ± 10.65 | | 64.03 ± 6.66 | | 67.69 ± 8.58 | | | | 67.23 ± 7.16 | | | | | 65.38 ± 14.03 | | |  |  |

| (a) Asymmetry of Swing Phase | (b) Pelvic Rotation | (c) Average Speed | (d) Average Left Stride Length | (e) Average Right Stride Length |
| --- | --- | --- | --- | --- |
| 1. Subject H1. | | | | |
| (a) Asymmetry of Swing Phase | (b) Pelvic Rotation | (c) Average Speed | (d) Average Left Stride Length | (e) Average Right Stride Length |
| 1. Subject H2. | | | | |
| (a) Asymmetry of Swing Phase | (b) Pelvic Rotation | (c) Average Speed | (d) Average Left Stride Length | (e) Average Right Stride Length |
| 1. Subject H3. | | | | |
| (a) Asymmetry of Swing Phase | (b) Pelvic Rotation | (c) Average Speed | (d) Average Left Stride Length | (e) Average Right Stride Length |
| 1. Subject H4. | | | | |
| (a) Asymmetry of Swing Phase | (b) Pelvic Rotation | (c) Average Speed | (d) Average Left Stride Length | (e) Average Right Stride Length |
| 1. Subject H5. | | | | |
| (a) Asymmetry of Swing Phase | (b) Pelvic Rotation | (c) Average Speed | (d) Average Left Stride Length | (e) Average Right Stride Length |
| 1. Subject H6. | | | | |
| (a) Asymmetry of Swing Phase | (b) Pelvic Rotation | (c) Average Speed | (d) Average Left Stride Length | (e) Average Right Stride Length |
| 1. Subject H7. | | | | |
| (a) Asymmetry of Swing Phase | (b) Pelvic Rotation | (c) Average Speed | (d) Average Left Stride Length | (e) Average Right Stride Length |
| 1. Subject H8. | | | | |
| (a) Asymmetry of Swing Phase | (b) Pelvic Rotation | (c) Average Speed | (d) Average Left Stride Length | (e) Average Right Stride Length |
| 1. Subject H9. | | | | |
| (a) Asymmetry of Swing Phase | (b) Pelvic Rotation | (c) Average Speed | (d) Average Left Stride Length | (e) Average Right Stride Length |
| 1. Subject H10. | | | | |

Figure S3. Experiments with animated stepping cues (healthy subjects).

Table S6. Performance indexes with animated stepping cues (healthy subjects)

|  | | *A* | | *B* | | | | *D* | | | | $\bar{B}$ | | | $\bar{A}$ | | | |
| --- | --- | --- | --- | --- | --- | --- | --- | --- | --- | --- | --- | --- | --- | --- | --- | --- | --- | --- |
| Asymmetry of the Swing Phase (%) (average ± standard derivation) | | | | | | | | | | | | | | | | | | |
| H1 | 15.54 ± 10.84 | | 12.25 ± 8.87 | | | 10.56 ± 7.58 | | | | 7.18 ± 4.69 | | | 11.61 ± 6.91 | | |  |  |  |
| H2 | 23.30 ± 21.21 | | 12.09 ± 10.27 | | | 11.81 ± 8.51 | | | | 11.46 ± 8.34 | | | 14.99 ± 10.67 | | |  |  |  |
| H3 | 9.98 ± 7.67 | | 9.14 ± 12.32 | | | 8.60 ± 7.16 | | | | 9.65 ± 8.84 | | | 9.74 ± 9.93 | | |  |  |  |
| H4 | 22.82 ± 13.46 | | 18.18 ± 7.63 | | | 17.24 ± 7.88 | | | | 16.27 ± 8.41 | | | 19.84 ± 8.71 | | |  |  |  |
| H5 | 31.07 ± 40.56 | | 29.56 ± 9.31 | | | 13.55 ± 6.27 | | | | 20.62 ± 6.43 | | | 16.95 ± 7.78 | | |  |  |  |
| H6 | 13.98 ± 9.05 | | 12.44 ± 9.49 | | | 11.58 ± 11.83 | | | | 12.07 ± 8.12 | | | 12.97 ± 7.14 | | |  |  |  |
| H7 | 16.79 ± 13.63 | | 13.46 ± 16.85 | | | 7.85 ± 5.44 | | | | 8.10 ± 5.90 | | | 9.86 ± 6.49 | | |  |  |  |
| H8 | 14.67 ± 7.51 | | 9.14 ± 7.21 | | | 7.65 ± 5.76 | | | | 8.90 ± 6.56 | | | 9.48 ± 6.36 | | |  |  |  |
| H9 | 19.43 ± 12.71 | | 11.99 ± 7.85 | | | 11.56 ± 7.17 | | | | 12.94 ± 8.43 | | | 15.16 ± 7.09 | | |  |  |  |
| H10 | 11.58 ± 6.80 | | 8.95 ± 7.28 | | | 10.71 ± 7.62 | | | | 9.93 ± 7.22 | | | 9.72 ± 6.28 | | |  |  |  |
| Amplitude of Pelvic Rotation (^o^) (average ± standard derivation) | | | | | | | | | | | | | | | | | |  |
| H1 | 8.41 ± 2.34 | | 8.65 ± 3.15 | | | | 13.39 ± 3.52 | | | | 13.09 ± 2.81 | | | 13.86 ± 2.17 | | |  |  |
| H2 | 15.61 ± 5.52 | | 12.68 ± 5.21 | | | | 10.42 ± 3.04 | | | | 9.68 ± 2.96 | | | 12.53 ± 3.23 | | |  |  |
| H3 | 3.34 ± 0.86 | | 3.34 ± 0.83 | | | | 4.31 ± 1.38 | | | | 4.78 ± 1.54 | | | 5.38 ± 1.46 | | |  |  |
| H4 | 10.87 ± 2.37 | | 10.25 ± 2.04 | | | | 11.23 ± 2.35 | | | | 11.93 ± 2.51 | | | 12.58 ± 2.06 | | |  |  |
| H5 | 19.13 ± 4.72 | | 17.83 ± 3.39 | | | | 8.11 ± 2.74 | | | | 12.32 ± 2.42 | | | 15.31 ± 3.34 | | |  |  |
| H6 | 9.77 ± 2.55 | | 13.45 ± 4.90 | | | | 23.02 ± 4.36 | | | | 17.60 ± 4.28 | | | 12.93 ± 4.87 | | |  |  |
| H7 | 5.37 ± 1.76 | | 5.45 ± 1.97 | | | | 5.84 ± 1.62 | | | | 6.91 ± 3.63 | | | 7.62 ± 1.99 | | |  |  |
| H8 | 10.82 ± 2.89 | | 15.12 ± 2.06 | | | | 13.27 ± 2.86 | | | | 13.07 ± 3.13 | | | 12.94 ± 1.98 | | |  |  |
| H9 | 6.67 ± 1.63 | | 9.54 ± 1.60 | | | | 9.71 ± 2.14 | | | | 10.22 ± 2.40 | | | 12.83 ± 2.90 | | |  |  |
| H10 | 6.45 ± 1.71 | | 9.05 ± 2.65 | | | | 7.17 ± 1.41 | | | | 7.81 ± 1.12 | | | 7.58 ± 0.88 | | |  |  |
| Walking speed (rpm) (average ± standard derivation) | | | | | | | | | | | | | | | | | |  |
| H1 | 335.99±0.0428 | | 446.63 ± 0.0381 | | 476.31 ± 0.0011 | | | | 476.31 ± 0.0013 | | | | | 476.31 ± 0.0013 | | |  |  |
| H2 | 183.58±0.1150 | | 468.60 ± 0.0331 | | 476.31 ± 0.0013 | | | | 476.31 ± 0.0012 | | | | | 463.18 ± 0.0534 | | |  |  |
| H3 | 331.69±0.0453 | | 464.38 ± 0.0362 | | 476.31 ± 0.0012 | | | | 476.31 ± 0.0013 | | | | | 476.31 ± 0.0013 | | |  |  |
| H4 | 179.03±0.1066 | | 457.22 ± 0.0634 | | 476.31 ± 0.0010 | | | | 476.31 ± 0.0010 | | | | | 464.60 ± 0.0057 | | |  |  |
| H5 | 315.96±0.0337 | | 385.35 ± 0.0791 | | 476.31 ± 0.0010 | | | | 476.31 ± 0.0010 | | | | | 476.31 ± 0.0012 | | |  |  |
| H6 | 157.02±0.0349 | | 334.70 ± 0.1389 | | 476.31 ± 0.0009 | | | | 476.31 ± 0.0009 | | | | | 476.31 ± 0.0009 | | |  |  |
| H7 | 235.97±0.0765 | | 356.81 ± 0.0584 | | 476.31 ± 0.0007 | | | | 476.31 ± 0.0008 | | | | | 476.31 ± 0.0007 | | |  |  |
| H8 | 313.12±0.1045 | | 470.84 ± 0.0115 | | 475.99 ± 0.0326 | | | | 439.54 ± 0.0312 | | | | | 476.31 ± 0.0013 | | |  |  |
| H9 | 325.83±0.0172 | | 458.55 ± 0.0472 | | 476.31 ± 0.0016 | | | | 476.31 ± 0.0016 | | | | | 476.31 ± 0.0014 | | |  |  |
| H10 | 249.01±0.1621 | | 476.04 ± 0.0026 | | 476.31 ± 0.0016 | | | | 476.31 ± 0.0015 | | | | | 476.31 ± 0.0015 | | |  |  |
| Stride length (cm) (average ± standard derivation) | | | | | | | | | | | | | | | | | | |
| H1 | 81.54 ± 11.59 | | 103.56 ± 6.99 | | 91.20 ± 3.33 | | | | 89.36 ± 4.38 | | | | | 87.96 ± 4.03 | | |  |  |
| H2 | 52.53 ± 14.79 | | 83.70 ± 8.30 | | 79.50 ± 5.28 | | | | 87.04 ± 3.88 | | | | | 86.53 ± 4.05 | | |  |  |
| H3 | 54.12 ± 4.79 | | 66.71 ± 4.17 | | 69.24 ± 3.11 | | | | 69.59 ± 3.54 | | | | | 64.70 ± 18.12 | | |  |  |
| H4 | 55.10 ± 9.61 | | 86.98 ± 9.61 | | 91.11 ± 10.79 | | | | 87.60 ± 11.97 | | | | | 91.73 ± 3.27 | | |  |  |
| H5 | 79.24 ± 3.46 | | 88.68 ± 10.56 | | 80.32 ± 6.15 | | | | 84.79 ± 2.52 | | | | | 83.65 ± 3.48 | | |  |  |
| H6 | 64.60 ± 26.33 | | 76.51 ± 11.28 | | 86.21 ± 8.79 | | | | 81.18 ± 4.25 | | | | | 83.57 ± 3.83 | | |  |  |
| H7 | 43.85 ± 11.75 | | 49.37 ± 7.00 | | 65.64 ± 40.20 | | | | 67.58 ± 8.49 | | | | | 65.16 ± 14.45 | | |  |  |
| H8 | 68.22 ± 14.46 | | 85.60 ± 16.88 | | 89.86 ± 22.05 | | | | 83.97 ± 18.36 | | | | | 79.75 ± 2.12 | | |  |  |
| H9 | 56.57 ± 5.47 | | 69.04 ± 5.51 | | 69.13 ± 4.95 | | | | 70.23 ± 2.58 | | | | | 70.76 ± 2.20 | | |  |  |
| H10 | 59.29 ± 11.40 | | 74.85 ± 2.72 | | 68.28 ± 9.13 | | | | 70.31 ± 8.58 | | | | | 71.64 ± 4.87 | | |  |  |

| (a) Asymmetry of Swing Phase | (b) Pelvic Rotation | (c) Average Speed | (d) Average Left Stride Length | (e) Average Right Stride Length |
| --- | --- | --- | --- | --- |
| 1. Subject S11. | | | | |
| (a) Asymmetry of Swing Phase | (b) Pelvic Rotation | (c) Average Speed | (d) Average Left Stride Length | (e) Average Right Stride Length |
| 1. Subject S12. | | | | |
| (a) Asymmetry of Swing Phase | (b) Pelvic Rotation | (c) Average Speed | (d) Average Left Stride Length | (e) Average Right Stride Length |
| 1. Subject S13. | | | | |
| (a) Asymmetry of Swing Phase | (b) Pelvic Rotation | (c) Average Speed | (d) Average Left Stride Length | (e) Average Right Stride Length |
| 1. Subject S14. | | | | |
| (a) Asymmetry of Swing Phase | (b) Pelvic Rotation | (c) Average Speed | (d) Average Left Stride Length | (e) Average Right Stride Length |
| 1. Subject S15. | | | | |
| (a) Asymmetry of Swing Phase | (b) Pelvic Rotation | (c) Average Speed | (d) Average Left Stride Length | (e) Average Right Stride Length |
| 1. Subject S16. | | | | |
| (a) Asymmetry of Swing Phase | (b) Pelvic Rotation | (c) Average Speed | (d) Average Left Stride Length | (e) Average Right Stride Length |
| 1. Subject S17. | | | | |
| (a) Asymmetry of Swing Phase | (b) Pelvic Rotation | (c) Average Speed | (d) Average Left Stride Length | (e) Average Right Stride Length |
| 1. Subject S18. | | | | |
| (a) Asymmetry of Swing Phase | (b) Pelvic Rotation | (c) Average Speed | (d) Average Left Stride Length | (e) Average Right Stride Length |
| 1. Subject S19. | | | | |
| (a) Asymmetry of Swing Phase | (b) Pelvic Rotation | (c) Average Speed | (d) Average Left Stride Length | (e) Average Right Stride Length |

(10) Subject S20.

Figure S4. Experiments with animated stepping cues (post-stroke patients).

Table S7. Performance indexes with animated stepping cues (stroke patients)

|  | | *A* | | *B* | | | | *D* | | | | $\bar{B}$ | | | $\bar{A}$ | | | |
| --- | --- | --- | --- | --- | --- | --- | --- | --- | --- | --- | --- | --- | --- | --- | --- | --- | --- | --- |
| Asymmetry of the Swing Phase (%) (average ± standard derivation) | | | | | | | | | | | | | | | | | | |
| S1 | 21.26 ± 7.39 | | 21.23 ± 10.93 | | | 20.42 ± 9.55 | | | | 19.24 ± 12.44 | | | 20.78 ± 11.09 | | |  |  |  |
| S2 | 19.52 ± 8.12 | | 12.90 ± 7.04 | | | 14.40 ± 7.20 | | | | 15.61 ± 9.03 | | | 18.21 ± 7.51 | | |  |  |  |
| S3 | 21.08 ± 11.98 | | 15.60 ± 13.47 | | | 15.40 ± 9.85 | | | | 17.60 ± 10.38 | | | 17.78 ± 11.14 | | |  |  |  |
| S4 | 22.19 ± 11.33 | | 18.62 ± 16.57 | | | 16.61 ± 8.58 | | | | 18.79 ± 9.96 | | | 16.57 ± 10.18 | | |  |  |  |
| S5 | 15.85 ± 8.75 | | 9.26 ± 8.13 | | | 8.59 ± 5.84 | | | | 9.26 ± 6.12 | | | 8.46 ± 5.60 | | |  |  |  |
| S6 | 31.26 ± 24.95 | | 18.97 ± 31.80 | | | 33.34 ± 21.91 | | | | 19.91 ± 15.55 | | | 18.28 ± 16.46 | | |  |  |  |
| S7 | 35.92 ± 13.50 | | 35.06 ± 13.24 | | | 30.45 ± 11.10 | | | | 30.69 ± 14.47 | | | 30.05 ± 9.75 | | |  |  |  |
| S8 | 27.34 ± 14.33 | | 25.88 ± 12.80 | | | 25.00 ± 14.40 | | | | 27.28 ± 16.12 | | | 26.47 ± 11.18 | | |  |  |  |
| S9 | 13.13 ± 9.86 | | 9.60 ± 8.15 | | | 8.33 ± 8.40 | | | | 8.81 ± 9.25 | | | 7.30 ± 8.03 | | |  |  |  |
| S10 | 39.75 ± 12.54 | | 35.76 ± 14.84 | | | 32.42 ± 9.58 | | | | 30.68 ± 8.51 | | | 30.94 ± 9.12 | | |  |  |  |
| Amplitude of Pelvic Rotation (^o^) (average ± standard derivation) | | | | | | | | | | | | | | | | | |  |
| S1 | 9.76 ± 4.27 | | 14.32 ± 2.11 | | | | 14.56 ± 1.63 | | | | 12.41 ± 1.78 | | | 12.25 ± 1.23 | | |  |  |
| S2 | 7.16 ± 0.91 | | 5.59 ± 1.21 | | | | 5.41 ± 0.94 | | | | 4.84 ± 0.91 | | | 6.02 ± 1.47 | | |  |  |
| S3 | 10.09 ± 3.75 | | 12.45 ± 2.50 | | | | 14.56 ± 2.09 | | | | 15.92 ± 2.20 | | | 17.41 ± 2.27 | | |  |  |
| S4 | 6.26 ± 1.56 | | 6.30 ± 1.71 | | | | 6.62 ± 1.29 | | | | 7.11 ± 1.26 | | | 6.69 ± 1.47 | | |  |  |
| S5 | 13.94 ± 2.05 | | 12.57 ± 4.86 | | | | 21.06 ± 5.93 | | | | 17.62 ± 3.90 | | | 14.98 ± 3.52 | | |  |  |
| S6 | 12.43 ± 1.87 | | 15.04 ± 2.45 | | | | 12.97 ± 2.60 | | | | 13.01 ± 2.19 | | | 12.94 ± 2.33 | | |  |  |
| S7 | 11.37 ± 4.20 | | 14.84 ± 1.94 | | | | 17.41 ± 1.51 | | | | 18.11 ± 2.14 | | | 13.53 ± 2.16 | | |  |  |
| S8 | 4.73 ± 1.36 | | 5.27 ± 1.48 | | | | 6.77 ± 3.10 | | | | 9.92 ± 1.50 | | | 10.02 ± 1.72 | | |  |  |
| S9 | 8.34 ± 2.02 | | 9.44 ± 2.10 | | | | 11.46 ± 1.46 | | | | 11.57 ± 1.60 | | | 10.48 ± 2.05 | | |  |  |
| S10 | 16.17 ± 4.65 | | 15.88 ± 2.38 | | | | 16.80 ± 1.39 | | | | 17.55 ± 1.58 | | | 15.65 ± 1.26 | | |  |  |
| Walking speed (rpm) (average ± standard derivation) | | | | | | | | | | | | | | | | | |  |
| S1 | 419.91±0.1583 | | 476.31 ± 0.0013 | | 476.31 ± 0.0013 | | | | 476.31 ± 0.0014 | | | | | 475.73 ± 0.0027 | | |  |  |
| S2 | 177.64±0.1366 | | 425.84 ± 0.0655 | | 476.31 ± 0.0015 | | | | 476.31 ± 0.0014 | | | | | 401.07 ± 0.0402 | | |  |  |
| S3 | 259.71±0.1363 | | 467.07 ± 0.0455 | | 476.31 ± 0.0018 | | | | 476.31 ± 0.0018 | | | | | 416.38 ± 0.0712 | | |  |  |
| S4 | 138.10±0.1039 | | 319.22 ± 0.0776 | | 382.42 ± 0.1715 | | | | 475.66 ± 0.0052 | | | | | 316.50 ± 0.1110 | | |  |  |
| S5 | 97.06 ± 0.0458 | | 362.05 ± 0.0503 | | 381.11 ± 0.0010 | | | | 381.11 ± 0.0011 | | | | | 381.10 ± 0.0012 | | |  |  |
| S6 | 141.41±0.0482 | | 215.29 ± 0.0137 | | 221.40 ± 0.0025 | | | | 222.25 ± 0.0007 | | | | | 222.31 ± 0.0005 | | |  |  |
| S7 | 203.71±0.1120 | | 360.00 ± 0.0503 | | 429.41 ± 0.0053 | | | | 476.31 ± 0.0020 | | | | | 460.64 ± 0.0287 | | |  |  |
| S8 | 131.87±0.0777 | | 334.54 ± 0.1075 | | 476.31 ± 0.0015 | | | | 476.31 ± 0.0015 | | | | | 476.31 ± 0.0015 | | |  |  |
| S9 | 443.47±0.0807 | | 476.31 ± 0.0017 | | 476.31 ± 0.0017 | | | | 476.31 ± 0.0017 | | | | | 476.31 ± 0.0016 | | |  |  |
| S10 | 129.73±0.0912 | | 212.13 ± 0.0745 | | 254.35 ± 0.0024 | | | | 283.95 ± 0.0034 | | | | | 249.54 ± 0.0018 | | |  |  |
| Stride length (cm) (average ± standard derivation) | | | | | | | | | | | | | | | | | | |
| S1 | 71.25 ± 8.01 | | 70.62 ± 7.60 | | 76.52 ± 11.17 | | | | 75.50 ± 24.47 | | | | | 95.10 ± 32.30 | | |  |  |
| S2 | 51.05 ± 5.28 | | 65.12 ± 5.60 | | 72.27 ± 3.42 | | | | 70.78 ± 2.41 | | | | | 66.26 ± 3.27 | | |  |  |
| S3 | 67.51 ± 20.14 | | 80.49 ± 6.58 | | 76.19 ± 8.09 | | | | 75.98 ± 4.84 | | | | | 68.83 ± 10.24 | | |  |  |
| S4 | 44.77 ± 16.44 | | 64.09 ± 13.59 | | 70.05 ± 19.30 | | | | 81.48 ± 14.32 | | | | | 60.06 ± 20.68 | | |  |  |
| S5 | 41.50 ± 18.13 | | 78.10 ± 14.41 | | 67.90 ± 8.55 | | | | 69.16 ± 40.46 | | | | | 94.77 ± 78.80 | | |  |  |
| S6 | 31.95 ± 5.11 | | 38.17 ± 3.21 | | 38.70 ± 7.78 | | | | 40.75 ± 5.00 | | | | | 40.54 ± 2.20 | | |  |  |
| S7 | 32.54 ± 12.11 | | 49.77 ± 14.88 | | 56.18 ± 2.71 | | | | 60.36 ± 8.34 | | | | | 59.49 ± 3.75 | | |  |  |
| S8 | 34.44 ± 18.99 | | 55.70 ± 21.83 | | 72.74 ± 15.06 | | | | 75.00 ± 10.54 | | | | | 73.14 ± 10.37 | | |  |  |
| S9 | 59.81 ± 15.85 | | 60.64 ± 12.17 | | 61.37 ± 9.70 | | | | 58.26 ± 12.12 | | | | | 61.40 ± 10.69 | | |  |  |
| S10 | 28.19 ± 15.84 | | 42.22 ± 14.40 | | 51.73 ± 9.41 | | | | 58.42 ± 14.05 | | | | | 56.16 ± 12.51 | | |  |  |
